# Homeostatic Regulation of Spike Rate within Bursts in Two Distinct Preparations

**DOI:** 10.1523/ENEURO.0259-24.2024

**Published:** 2024-09-03

**Authors:** Alishah Lakhani, Carlos Gonzalez-Islas, Zahraa Sabra, Nicholas Au Yong, Peter Wenner

**Affiliations:** ^1^Department of Cell Biology, Emory University School of Medicine, Atlanta, Georgia 30322; ^2^Doctorado en Ciencias Biológicas Universidad Autónoma de Tlaxcala, Tlaxcala 90070, México; ^3^Department of Neurosurgery, Emory University School of Medicine, Atlanta, Georgia 30322

**Keywords:** chick embryo, cortical culture, homeostatic plasticity, multielectrode array, spinal cord

## Abstract

Homeostatic plasticity represents a set of mechanisms thought to stabilize some function of neural activity. Here, we identified the specific features of cellular or network activity that were maintained after the perturbation of GABAergic blockade in two different systems: mouse cortical neuronal cultures where GABA is inhibitory and motoneurons in the isolated embryonic chick spinal cord where GABA is excitatory (males and females combined in both systems). We conducted a comprehensive analysis of various spiking activity characteristics following GABAergic blockade. We observed significant variability in many features after blocking GABA_A_ receptors (e.g., burst frequency, burst duration, overall spike frequency in culture). These results are consistent with the idea that neuronal networks achieve activity goals using different strategies (degeneracy). On the other hand, some features were consistently altered after receptor blockade in the spinal cord preparation (e.g., overall spike frequency). Regardless, these features did not express strong homeostatic recoveries when tracking individual preparations over time. One feature showed a consistent change and homeostatic recovery following GABA_A_ receptor block. We found that spike rate within a burst (SRWB) increased after receptor block in both the spinal cord preparation and cortical cultures and then returned to baseline within hours. These changes in SRWB occurred at both single cell and population levels. Our findings indicate that the network prioritizes the burst spike rate, which appears to be a variable under tight homeostatic regulation. The result is consistent with the idea that networks can maintain an appropriate behavioral response in the face of challenges.

## Significance Statement

Homeostatic plasticity plays a critical role in maintaining optimal neural function, particularly during development when the system undergoes repeated functional challenges. In our current study, GABA receptor activity was blocked in two different systems, one in which GABA is inhibitory and another in which GABA is excitatory. In both, we observed that the spike rate within a burst (SRWB) consistently increased and homeostatically returned to control levels in the continued presence of the blocker, demonstrating the importance of SRWB maintenance. When a network is called into action or is functionally engaged during a synaptic barrage, a critical feature that is homeostatically maintained is the spike rate during this activity, which would be crucial for network behavioral performance.

## Introduction

Neuronal activity patterns can be highly stereotyped and invariant, even though the ion channels and synaptic inputs that drive such activity can vary widely (Liu et al., 1998; Prinz et al., 2004; Hamood and Marder, 2014; Goaillard and Marder, 2021). For instance, in the crab stomatogastric ganglia, neurons expressing the same activity pattern can show a threefold variation in channel conductances (Schulz et al., 2006; Hamood and Marder, 2014). This variation arises as cells homeostatically maintain some feature of activity but achieve this in different ways (degeneracy). Because of this variability, an activity perturbation will affect one cell/network differently than another, as some are more dependent on certain conductances/synapses (Hamood and Marder, 2014; Sakurai et al., 2014; Hamood et al., 2015). Both this variability and the activity feature that is homeostatically maintained can be observed by perturbing network activity and observing how different activity features are altered and homeostatically recover.

However, identifying the actual neural characteristics that are homeostatically maintained has been elusive. Different studies have identified different homeostatic goals. Studies have found evidence supporting overall firing rate homeostasis at the level of the individual cell (Hengen et al., 2013, 2016), at the network population level (Slomowitz et al., 2015), in response to a sensory stimulus (Kuhlman et al., 2013), and in terms of criticality that maximizes information capacity (Ma et al., 2019). Due to the importance of homeostatic plasticity in neural disorders, it is critical that we come to a better understanding of the multiple activity properties that may be homeostatically regulated.

With the idea that individual preparations achieve similar activity using different strategies and that different spiking features are homeostatically regulated, our goal was twofold. First, we assessed variability of firing properties in individual preparations following a synaptic perturbation. Second, we examined which properties were best homeostatically regulated (Buzsáki et al., 2002; Niemeyer et al., 2021) and have done this using two very different developing systems.

We and others have demonstrated that the addition of CNQX, an AMPA receptor (AMPAR) antagonist, to cortical cultures results in a wide range of changes in overall firing rates and that homeostatic recovery of the spike rates did not occur within 24 h (Fong et al., 2015; Slomowitz et al., 2015). In the current study, we blocked GABA_A_ receptor (GABAR) activation in cortical cultures. Our primary objective was to identify the specific spiking features that undergo homeostatic regulation in response to perturbation. To assess the generality of our observations in culture in a more intact system, we also chose the isolated embryonic chick spinal cord preparation. This system expresses several homeostatic plasticity mechanisms, both *in vivo* and in vitro (Gonzalez-Islas and Wenner, 2006; Wilhelm and Wenner, 2008; Wenner, 2020; Pekala and Wenner, 2022). Previous work has suggested that GABAR blockade in ovo, during a developmental stage when GABA is depolarizing and excitatory, leads to the abolition of embryonic movements that are then recovered 12 h after drug application (Wilhelm and Wenner, 2008). Nevertheless, the precise features of activity that were homeostatically regulated in motoneurons were unknown. Thus, the current study aimed to understand how activity features are altered and homeostatically restored in response to GABAR blockade in two systems: one in which GABA is inhibitory (cortical culture) and the other in which GABA is excitatory (isolated spinal cord).

Following GABAergic blockade, we assessed overall spike rate, burst frequency and duration, spike rate within a burst (SRWB), and interburst spike rate. As predicted due to degeneracy, we observed variability in both preparations after pertuirbation. On the other hand, we found that SRWB was most consistently altered (increased) and was also most reliably returned to preperturbation levels in both systems. Our findings suggest that both cells and the network actively and homeostatically restore spike rate during synaptic bursts, thus preventing long-term hyperexcitability.

## Materials and Methods

### Cell cultures

Brain cortices were obtained from C57BL/6J embryonic day 17 (E17) mice from BrainBits (unknown sex). Neurons were obtained after the cortical tissue was enzymatically dissociated with papain. Cell suspension was diluted to 2,500 live cells per microliter, and 35,000 cells were plated on planar multielectrode arrays (MEAs) coated with poly-d-lysine (Sigma, P-3143) and laminin. The cultures were maintained in Neurobasal medium supplemented with 2% B27 and 2 mM GlutaMAX. No antibiotics or antimycotics were used. Medium was changed completely after one day in vitro (1 DIV) and half of the volume was then changed every 7 d. Spiking activity was monitored starting ∼10 DIV to determine if a bursting phenotype was expressed and continuous recordings were made between 14 and 20 DIV. Cultures were discarded after 20 DIV. All protocols followed the National Research Council's Guide on regulations for the Care and Use of Laboratory Animals and from the Animal Use and Care Committee.

### Spinal cord dissection

Experiments were performed on White Leghorn chicken embryos (Hy-Line North America, unknown sex) at E11 (or Stage 37; Hamburger and Hamilton, 1951). The spinal cords were isolated at E11, with ventral roots attached, in cooled (14°C) and oxygenated (95% O_2_/5% CO_2_) Tyrode's solution containing the following (in mM): 139 NaCl, 12 d-glucose, 17 NaHCO_3_, 3 KCl, 1 MgCl_2_, and 3 CaCl_2_ (Pekala and Wenner, 2022). After dissection, the cord was left overnight to recover in Tyrode's solution at 18°C. The cord was then transferred to a recording chamber and continuously oxygenated in Tyrode's solution that was warmed up to 27 ± 1°C and contained 5 mM KCl rather than the 3 mM used in the dissecting Tyrode's solution, which produced more consistent episodes of spontaneous network activity (SNA).

### Electrophysiology recordings

#### Culture

Extracellular spiking was recorded from cultures plated on a planar 64-channel MEA (Multi Channel Systems) recorded between 14 and 20 DIV. MEAs were placed in the MZ60 headstage (Tucker-Davis Technologies—TDT), which was housed in a 5% CO_2_ incubator at 37°C. Drugs were added separately in a sterile hood and then returned to the MEA recording system. Recordings were bandpass filtered between 200 and 3,000 Hz and acquired at 25 kHz sampling rate. For bicuculline-treated cultures, 20 µM bicuculline was added to the culture following 2–3 h of baseline activity recordings.

#### Spinal cord

Ventral roots were drawn into suction electrodes, and motoneurons were identified by antidromically stimulating the ventral root at 30 µA with an ISO-Flex stimulator isolator. Extracellular recordings were made using silicon probes (A1×32-Poly2-5mm-50s-177, NeuroNexus) with 32 recording sites covering 775 μm in depth. The probe was inserted at a 30° angle (lateral to medial) into the upper lumbar region of the isolated spinal cord from the ventral side. Recordings were bandpass filtered between 300 and 2,000 Hz and acquired at 25 kHz. For gabazine-treated cords, 10 µM gabazine was added to the bath after recording baseline activity for ∼1 h. Synapse software (Tucker-Davis Technologies) was used to monitor activity on a TDT electrophysiological platform consisting of the PZ2 preamplifier and the RZ2 BioAmp Processor for both preparations.

### Calcium imaging

Calcium indicator Calcium Green-1 dextran (10,000 MW) was used to retrogradely label motoneurons overnight as described previously (O'Donovan et al., 2005). After isolation of the embryonic chick spinal cord (see above), lumbosacral spinal nerves were drawn into a suction electrode. Saline was then withdrawn from the suction electrode and replaced by a concentrated solution of Calcium Green (1 mg in 10 µl of H_2_O). The indicator then retrogradely labeled motoneuron somata overnight. The next morning, the pia was removed in the area of the labeled motoneurons, and the preparation was moved to a chamber and placed ventral side-down on a coverslip. Images were continuously acquired of labeled motoneurons through the ventral white matter using an inverted microscope (Olympus IX70) via an intensified charge-coupled detector video camera (Stanford Photonics). The tissue was illuminated using a 75 W xenon arc lamp with an excitation filter of 450–490 nm, dichroic of 510 nm, and a barrier filter of 520 nm. Various ND filters were used to reduce photodynamic damage. During the experiment, video data (7–15 frames per second) were acquired and stored using SimplePCI. Images were then processed and analyzed using Fiji software (WS Rasband, ImageJ, National Institutes of Health; http://rsb.info.nih.gov/ij/; 1997–2006). To record calcium transients during episodes of SNA, spinal nerves were stimulated. Video recordings were ∼2 min in length. During episodes of network activity, virtually all labeled neurons showed changes in fluorescence or became optically active (O'Donovan et al., 1994). An average image was then made in order to draw regions of interest (ROIs) over individually labeled motoneurons. The change in fluorescence was monitored by measuring average pixel intensity for 10 ROIs per spinal cord in three different cords. To assess the change in fluorescence as a percentage of the baseline fluorescence of a motoneuron, we measured fluorescence by taking the ROI value of each frame and subtracting the average value of 30 frames before the episode of SNA. We then took the change in fluorescence of each ROI and divided it by the average before the episode minus an ROI in a nonlabeled region (autofluorescence) ((ROI − ROI average before episode) / (ROI average before episode − autofluorescence)).

### Data analysis

Spiking activity from cortical cultures and isolated spinal cords was analyzed offline with a custom-written Matlab program (Gonzalez-Islas et al., 2024). The recordings (acquired in TDT) were subsequently converted using the function TDTbin2mat to Matlab files (MathWorks). A custom-written Matlab program identified bursts of network spikes using an interspike interval–threshold detection algorithm as described previously, where “A burst was identified if *N* spikes occurred in less than *T* ms, where the threshold *T* was automatically determined from observing the probability distribution of inter-spike-intervals.” The number of spikes comprising the smallest burst was considered *N* (Bakkum et al., 2013). The program plotted the probability distribution for the periods between 10 consecutive spikes. The longest duration minima were chosen as the threshold duration (*T*; Extended Data Fig. 1-1*A*) to find a user-defined number of spikes (*N*) within a burst as described previously (Bakkum et al., 2013). Spiking activity was labeled as a network burst when it met a user-defined minimum number of spikes across all channels (culture, 10; cord, 4–8) occurring across a user-defined minimum number of channels (culture, 5–10; cord, 3–5). We removed channels that did not contribute to bursts or were constantly active (potential noise). For example, in Extended Data Figure 1-1*A*,we plotted the interval between the 1st and 10th consecutive spike and saw the largest minima (dip in the probability distribution) was ∼200 ms. We then used this value to search for periods where 10 spikes occurred within 200 ms and then repeat this process after advancing one spike at a time (e.g., spike 1–10 occur within 200 ms and then determine if spike 2–11 is still within 200 ms—if so, the identified burst is lengthened). We then visually assessed the bursts identified by the program [Extended Data Fig. 1-1*B*; the duration of the identified bursts is shown as a red line below the burst and spike times can be seen within the burst (red) and outside the burst (black)]. The program also automatically computed network burst metrics including burst frequency, overall spike rate, and other characteristics. An additional feature of the program is its ability to identify and calculate characteristics of SNA in the isolated spinal cord. SNA presents as a series of bursts within 30–60 s (called an episode), followed by a 5–10 min quiescent period. Spiking activity was labeled as an episode when it met user-defined criteria. This included episode duration (typically 20–100 s). The episodes were then visually inspected to ensure that the chosen parameters accurately identified episodes and the bursts within the episodes. The program also computed episode metrics, such as the number of spikes, duration, and channels involved.

#### Culture

Each time point (0, 0.5, 1, 3, 6, 9, 12, 18, 24 h) indicated in the figures represents recordings acquired in a 15 min interval (900 s), where multiple features of spiking were calculated. Overall spike rate was defined as the total number of spikes across all channels divided by the duration of that recording period (total number of spikes/900 s). The burst frequency was calculated as the number of bursts identified in a recording session divided by the total duration of that recording session (total number of bursts/900 s). The interburst interval (IBI) spike rate was calculated as the number of spikes that occur outside of bursts divided by the total IBI (combined durations of all interburst periods) in the 900 s recording session. Average burst duration was the average duration of all bursts in a recording session. SRWB was calculated in two different ways—line graphs in the main figures show the total number of spikes within all bursts divided by the sum of the burst durations, or in the extended data, line graphs are calculated by averaging all SRWBs calculated for each burst, thus providing standard deviation for each time point within a culture. Due to the mentioned variability between cultures, the line graphs of the main figures show the data normalized to baseline. Additionally, in the line graphs of the extended data, we show raw data (not normalized). Homeostatic recovery of spiking features (homeostatic index) was computed by first calculating the recovery in bicuculline-treated cultures (maximum normalized value within the first hour in bicuculline minus the normalized value at the 24 h time point in bicuculline). This number was then divided by the maximum normalized value within the first hour in bicuculline minus the normalized value in control cultures at 24 h (typically near 1). The homeostatic index was calculated and is displayed for each culture separately (Fig. 4, individual dots).

#### Spinal cord

Spiking activity was divided into 30 min bins, and only spiking in motoneuron channels, as determined by antidromic ventral root stimulation, was included in the analyses. Within each time bin, the following characteristics were calculated: episode duration, episode spike rate, burst duration, SRWB, interepisode interval spike rate, and overall spike rate. Episode duration was calculated as the time between the onset of the first burst and the end of the last burst. Episode spike rate is the number of spikes in motoneuron channels divided by the episode duration. Burst duration is the average burst period within the episodes. SRWB is the total number of spikes across motoneuron channels that occur during bursts within the episode divided by the total duration of the bursts (spikes that occurred in the episode but outside of the bursts were not included). If more than one episode occurred during the 30-minute bin, the metrics were averaged so that only one value for each spinal cord is displayed. In addition, in each 30 min window, the overall spike rate (as described above) and the interepisode interval spike rate (number of spikes outside of episodes divided by the interepisode time window) were calculated. Since each isolated spinal cord had different baseline values, all data shown are normalized to baseline (30 min of recording before drugs).

### Statistical analysis

Estimation statistics have been used throughout the manuscript. Five thousand bootstrap samples were taken; the confidence interval is bias corrected and accelerated. The *p* values reported are the likelihoods of observing the effect sizes, if the null hypothesis of zero difference is true. For each permutation *p* value, 5,000 reshuffles of the control and test labels were performed [moving beyond *p* values: data analysis with estimation graphics (Ho et al., 2019)].

### Data availability

Analyzed data values and the code for the Matlab program that detects burst features can be found at https://github.com/pwenner/eNeuro-2024.

## Results

### SRWB is homeostatically restored following GABAergic blockade in cortical cultures

**Figure 1.**
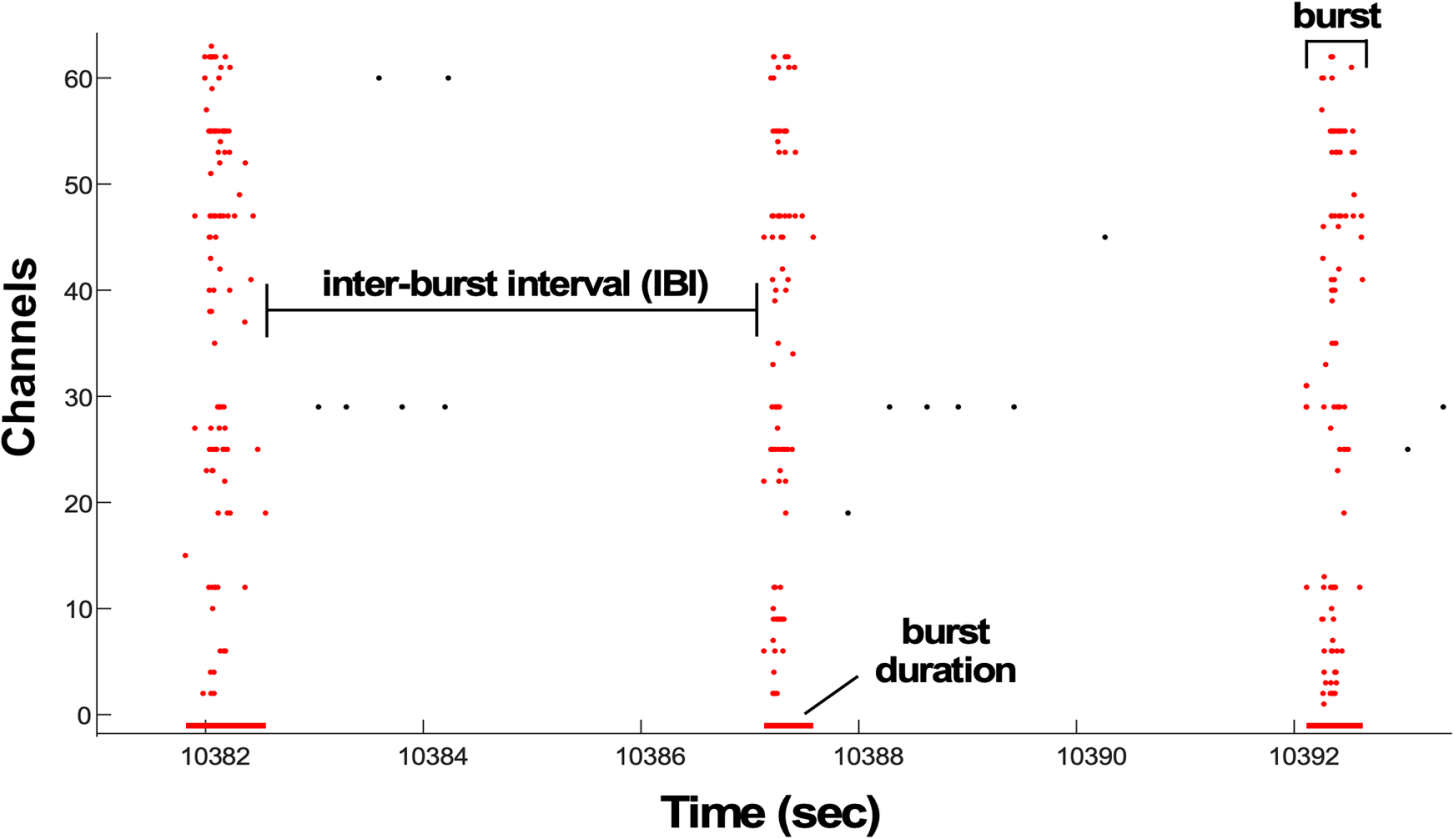
Firing rate characteristics analyzed in cortical cultures. Raster plot of network burst activity across all channels in the MEA. Interburst interval (IBI) spike rate is the spiking that occurred outside of the bursts (spikes are black dots). SRWB is the spike rate that occurs within the burst (spikes are red dots). Burst duration is shown by the red lines below raster plot. Extended Data Figure 1-1 provides more details on the program that analyzes bursts.

Previous work has shown that AMPA receptor blockade (CNQX) in cortical cultures changed the firing rates and burst frequencies in a highly variable manner (Fong et al., 2015). Both the degree to which firing/burst rates were reduced and the 24 h homeostatic recovery were variable in these different cultures. We wondered if such variability would similarly be observed if we attempted to increase activity. GABAR blockade in cortical cultures has been shown to increase certain features of spiking acutely (Turrigiano et al., 1998; Corner et al., 2002; Weihberger et al., 2013). In order to assess the variability of this response and to determine the timing of such a recovery, we disinhibited cortical cultures and monitored various firing rate characteristics following this perturbation. To this end, we plated mouse cortical cultures on 64-channel MEAs and allowed for circuit development for 10–14 DIV. We observed typical network burst activity, with bursts involving relatively synchronous spikes across multiple channels in semiregular intervals. Then, we analyzed overall spike rate, burst duration and frequency, IBI spike rate (IBI, spike rate outside of bursts), and SRWB (Fig. 1). Baseline activity was recorded for several hours before the drug was introduced, and spiking features were then monitored for 24 h after the addition of the GABAR antagonist bicuculline (20 µM) or in untreated control cultures.

10.1523/ENEURO.0259-24.2024.f1-1 Figure 1-1 Program that detects and analyzes bursts. A) Probability distribution of ISI durations between 10 consecutive spikes (n to n + 9) largest dip in the distribution (200  ms) is used to detect bursts in the burst detection subroutine of the program. B) Bust detection program identifying bursts that contain at least 10 spikes in 200  ms. Burst duration is shown in red line below raster plot. Burst spikes are red and spikes in the inter-burst interval are black. Download Fig 1-1, TIF file.

#### Overall spike rate

**Figure 2.**
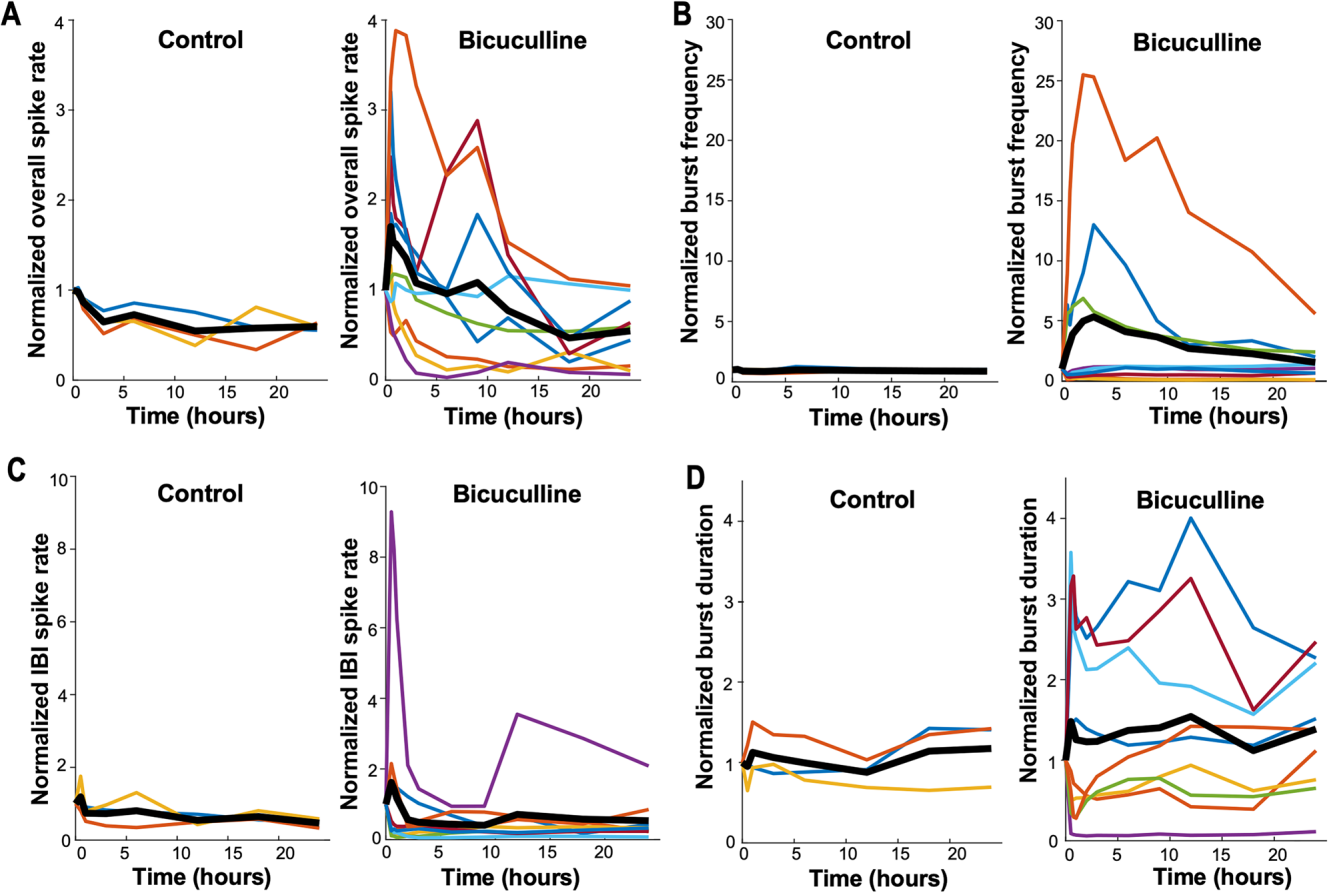
Burst dynamic parameters measured following GABAR blockade (bicuculline) in cortical cultures. ***A***, Normalized overall spike rate, (***B***) normalized burst frequency, (***C***) normalized interburst interval (IBI) spike rate and (***D***) normalized burst duration displayed over a 24 h period for control and bicuculline-treated cultures. Values at each time point are normalized to baseline (predrug) condition. Each color line represents a single culture, with the thick black line representing the mean of all cultures. The data shown in ***A*** and ***B*** are the same data as in our previous publication (Gonzalez-Islas et al., 2024); however, here we follow individual cultures over time. Estimation statistics comparing bicuculline-treated and control cultures at each of the time points establish that none are significantly different. This is likely due to the dramatic variability. Extended Data Figure 2-1 provides estimation statistics for data shown in Figure 2.

Previously, it was observed that overall spike rate increased shortly after bicuculline addition, although it did not reach significance (Gonzalez-Islas et al., 2024). Here, we tracked overall spiking across individual cultures over a 24 h period. On average (Fig. 2*A*, thick black line), this parameter increased following bicuculline, and then 24 h after GABAergic blockade, it had recovered to the level of control cultures at 24 h. However, looking at individual cultures tells a different story, as we observed strikingly distinct responses to bicuculline in each culture (Fig. 2*A*, different colors). While most cultures showed a clear increase in overall spiking, one briefly increased and then was reduced, one was unchanged, and two others were profoundly reduced. By 24 h, cultures appeared to be above, below, or at untreated control levels (Fig. 2*A*). The two cultures that were reduced from the onset of bicuculline remained low for the entire 24 h (showed no homeostatic tendency). Consistent with degeneracy, it was clear that following disinhibition, there was dramatic variability in both overall spike rate and the homeostatic recovery of this feature. In addition, prebicuculline values (not normalized) for all parameters are shown in Table 1.

10.1523/ENEURO.0259-24.2024.f2-1 Figure 2-1 Estimation statistics of burst dynamic parameters from bicuculline-treated cultures for A) overall spike rate B) burst frequency C) inter-burst interval (IBI) spike rate and D) burst duration. The mean differences at each time point are compared to control and displayed in Cumming estimation plots. Upper panel shows raw data from recordings of individual cultures (filled circles), where the mean value is represented by the gap in the vertical bars and the SD is represented by the vertical bars. Lower panel shows mean differences between control and treated groups as a bootstrap sampling distribution (mean difference is represented by filled circle and the 95% CIs are depicted by vertical error bars). Download Fig 2-1, TIF file.

**Table 1.** Mean and standard deviation of all spiking activity features that were analyzed for culture preparations

Feature	Prebicuculline value
Overall spike rate	42.51 ± 55.36
Burst frequency	0.24 ± 0.24
Interburst interval spike frequency	8.14 ± 8.21
Burst duration	1.07 ± 2.23
Spike rate within a burst	166.27 ± 76.46
Number of channels in a burst	16.24 ± 14.11

All features are reported in Hz, except for burst duration (seconds) and the number of channels.

#### Burst frequency/IBI spike rate

Since most spikes occurred within bursts, we also assessed burst frequency. Previously, it was found that burst frequency increased following bicuculline application, although it did not reach significance (Gonzalez-Islas et al., 2024). Here, we show burst frequency tracked across individual cultures over a 24 h period and found that burst frequency showed an apparent homeostatic nature if one only looked at the average values following bicuculline treatment (Fig. 2*B*, thick black line). However, once again, this view was a simplistic one as the dramatic variability to this perturbation complicated such an interpretation. Unlike overall spike rate, most cultures reduced burst frequency after GABAergic blockade (Fig. 2*B*). On the other hand, three of the cultures showed very large increases in burst frequency, which did appear to be in the process of recovering back toward control levels, although they did not fully recover by 24 h. The cultures that expressed reduced burst frequency following bicuculline either returned to control levels or were maintained at lower levels. Ultimately, we found that cultures demonstrated high variability and not all demonstrated a homeostatic recovery of burst frequency. Very similar results were observed when looking at the spike rate across channels in the IBI (most decreased, some increased, no obvious homeostatic response; Fig. 2*C*, Extended Data Fig. 2-1*C*).

#### Burst duration

We also analyzed burst duration (the period of synchronous spiking across channels) and found dramatic variability in burst duration following GABA_A_ receptor blockade (Fig. 2*D*, Extended Data Fig. 2-1*B*). Some cultures increased burst duration, some were unchanged, and some were profoundly decreased. However, whether there was an increase, decrease, or no change in burst duration, the postbicuculline value was largely maintained for the 24 h treatment. The majority of cultures did not demonstrate homeostasis of burst duration and responded to bicuculline in a highly variable manner.

#### Spike rate within a burst

**Figure 3.**
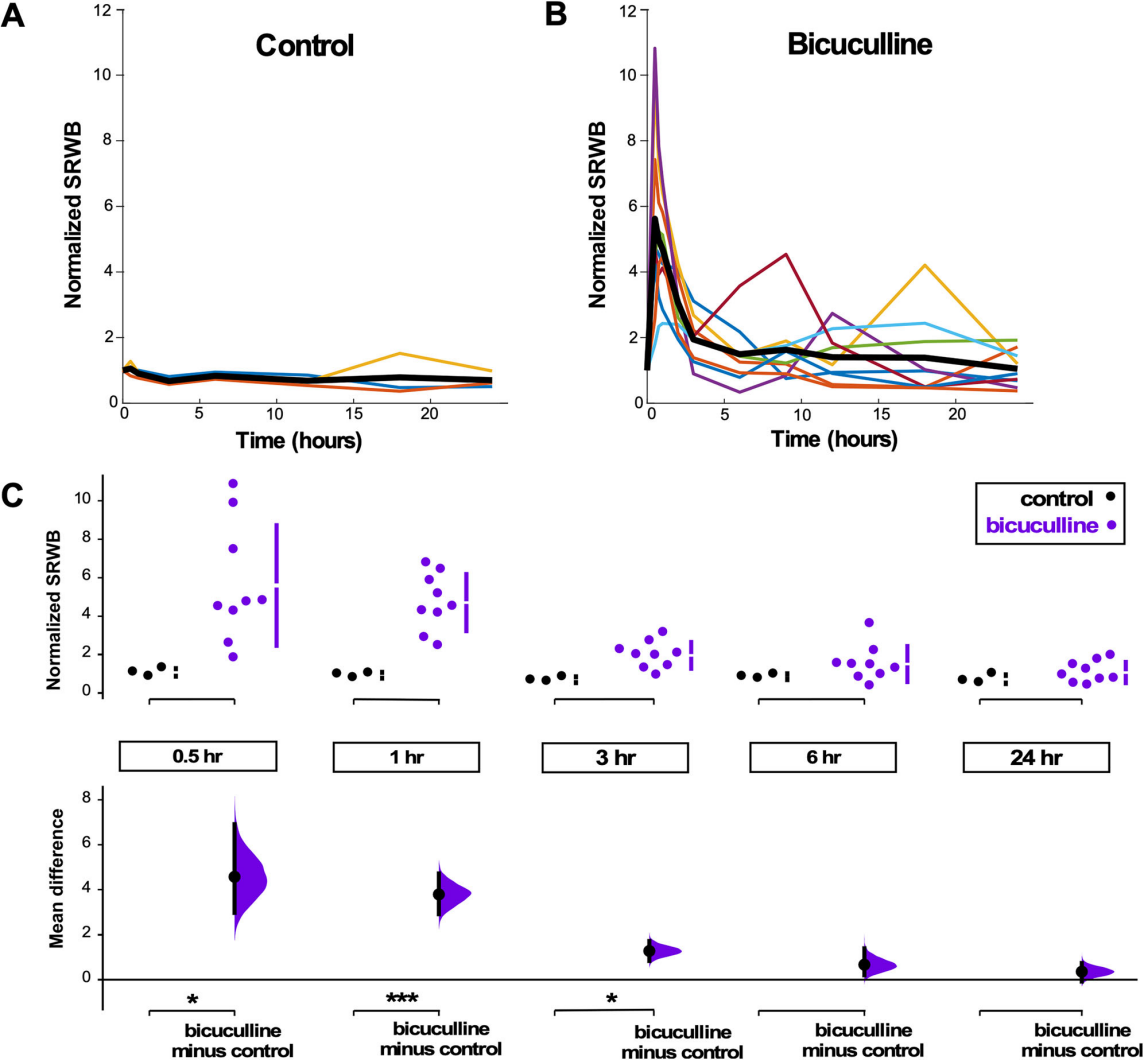
Spike rate within a burst (SRWB) following GABAR blockade is consistently homeostatically recovered in cortical cultures. ***A***, ***B***, SRWB displayed over a 24 h period for (***A***) control (untreated) and (***B***) bicuculline-treated cultures. Values at each time point are normalized to baseline (predrug) condition. Each color line represents a single culture, with the thick black line representing the mean of all cultures. ***C***, SRWB is compared for control and bicuculline-treated cultures at 0.5, 1, 3, 6, and 24 h after addition of bicuculline. The mean differences at different time points are compared with control and displayed in Cumming estimation plots. Significant differences denoted by *p < 0.05, ***p < 0.001. The top panel shows raw data from single culture recordings (filled circles), where the mean value is represented by the gap in the vertical bars and the SD is represented by the vertical bars. The bottom panel shows mean differences between control and treated groups as a bootstrap sampling distribution (mean difference is represented by filled circle and the 95% CIs are depicted by vertical error bars). Extended Data Figure 3-1 graphs non-normalized values for each culture individually. Extended Data Figure 3-2 shows non-normalized values for three cultures treated with gabazine instead of bicuculline.

We analyzed the SRWB, or the total number of spikes across all channels within a burst divided by the total duration of the bursts (Fig. 3). Within the first 30 min of disinhibition, there was a significant and dramatic increase in the SRWB for all cultures, and this effect persisted to some extent until approximately the 6th hour after bicuculline application, when the values homeostatically returned to levels seen before bicuculline was added and to levels seen in the untreated cultures at the corresponding time points. The first 3 h of bicuculline-treated cortical cultures demonstrate a significant increase in SRWB, while the 6 and 24 h time points were no different than control cultures (Fig. 3*C*). This result was remarkable, and so we further analyzed this interesting finding. We examined individual SRWBs for each burst in an individual culture and plotted the average value and standard deviation of this parameter and did so without normalization. It was clear that following bicuculline, SRWB was increased and homeostatically recovered within several hours of drug application in every single culture (Extended Data Fig. 3-1). Finally, bicuculline has been reported to modulate potassium channel conductances (Khawaled et al., 1999), so we also tested a weaker but more specific GABA receptor antagonist gabazine (5 µM) in three additional cultures. The results were no different than those using bicuculline (Extended Data Fig. 3-2). These striking results suggested that the blockade of GABAergic transmission triggered a uniform increase in SRWB that was then homeostatically brought back down to control levels.

10.1523/ENEURO.0259-24.2024.f3-1 Figure 3-1 Nine different cortical cultures show that addition of bicuculline triggers an increase in SRWB, which then is homeostatically returned to baseline levels. SRWB is calculated as the average SRWB across individual bursts and standard deviation of SRWB is shown as error bars. Data is not normalized and therefore represents the MEA-wide spike rate within a burst of each culture before (black) and after bicuculline (red). Download Fig 3-1, TIF file.

10.1523/ENEURO.0259-24.2024.f3-2 Figure 3-2 Three different cortical cultures show that addition of gabazine triggers an increase in SRWB, which then is homeostatically returned to baseline levels. SRWB is calculated as the average SRWB across individual bursts and standard deviation of SRWB is shown as error bars. Data was not normalized and therefore represents the MEA-wide spike rate within a burst for each culture before (black) and after gabazine (red). Download Fig 3-2, TIF file.

#### Homeostatic index of spiking features

**Figure 4.**
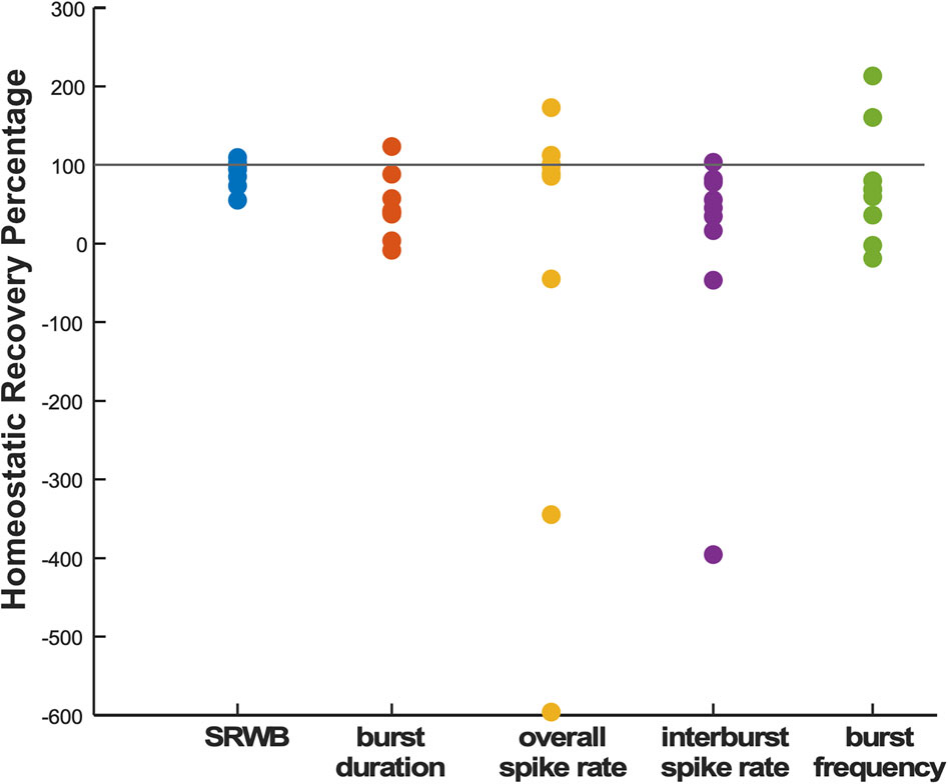
Homeostatic recovery percentage for different firing rate properties. The homeostatic index for each bicuculline-treated culture was calculated by dividing the actual recovery by the full potential recovery (see text). Horizontal line represents 100% recovery. Spike frequency within a burst is the firing rate property that most consistently demonstrates near 100% homeostatic recovery.

To better analyze and compare the homeostatic nature of each firing rate property, we assessed a homeostatic index (Fig. 4). We calculated this index to provide a percentage recovery of the perturbed state. This was calculated as the most extreme normalized value in the first hour after bicuculline minus the normalized value 24 h after bicuculline (actual recovery); this value was then divided by the most extreme normalized value in the first hour after bicuculline minus the normalized value in control cultures at 24 h (full recovery). For example, if the parameter increased to five times the initial value within the first hour and then returned to twice the 24 h control value, then the index would show a 75% recovery (5-2/5-1). In this way, values that do not recover at all are 0%, those that recover completely are at 100%, those above 100% recover but then continue past baseline control values, and those that are negative have continued to move in the direction of the first hour. We found that there was high variability in terms of homeostatic recovery for most of the firing rate properties we measured. However, for each one of the individual cultures, SRWB was the one property that most consistently returned to control levels (100%) and was associated with the smallest variability (coefficient of variation or CV; Fig. 4, Table 2). These studies demonstrate a critical importance of this firing rate property that only occurs when the network is synaptically active.

**Table 2.** Coefficient of variation (CV) and mean for homeostatic indices of all spiking activity features that were analyzed for culture preparations and shown in Figure 4

Feature	CV	Mean
SRWB	0.19	90.32
Burst duration	0.86	46.65
Overall spike rate	−7.12	−36.56
Interburst interval spike rate	−46.22	−3.32
Burst frequency	0.99	73.75

All features are reported in Hz, except for burst duration and episode duration (seconds).

#### SRWB is homeostatically regulated at both the population and single cell levels

**Figure 5.**
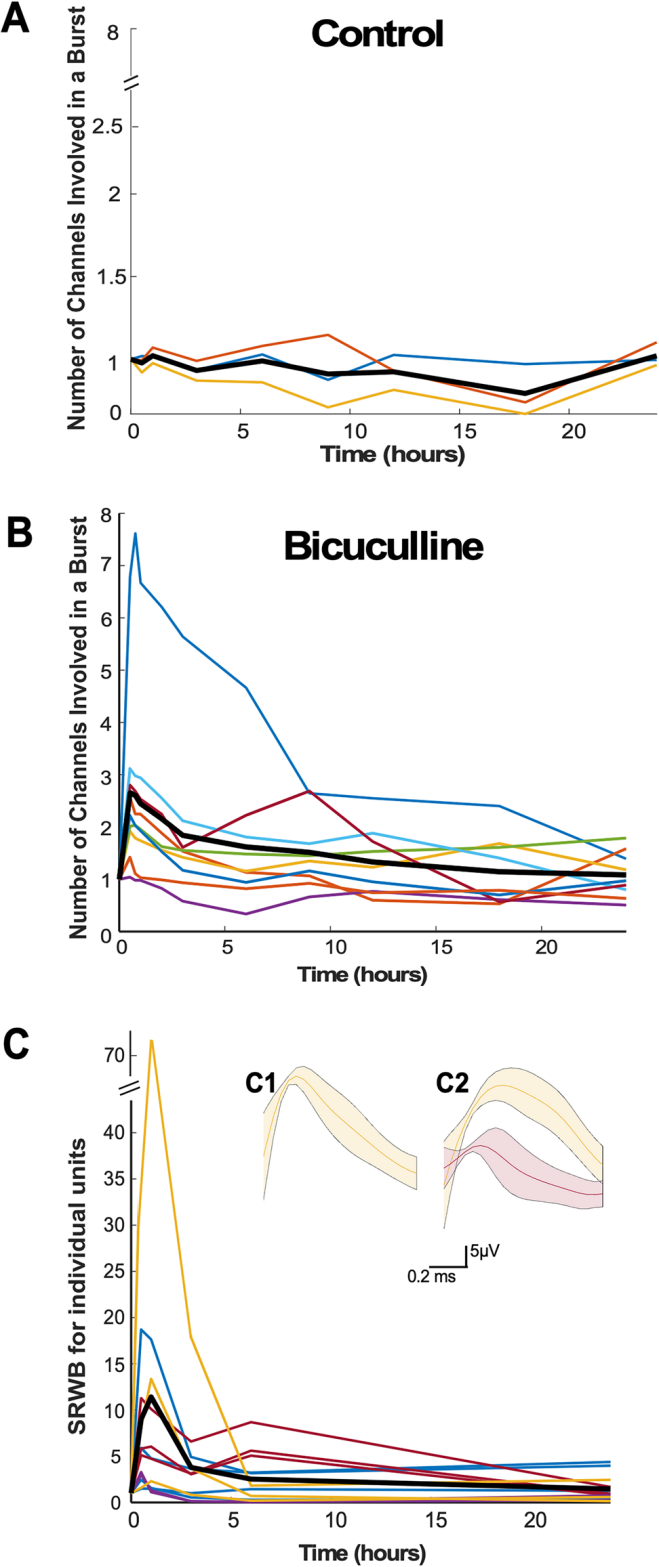
Spike rate within a burst (SRWB) following GABAR blockade was homeostatically recovered at both the population and single-unit level. ***A***, In control cultures, the number of MEA channels contributing to a burst remained stable. ***B***, Following bicuculline, the number of MEA channels contributing to a burst increased and was then homeostatically restored over a 24 h period. Values at each time point were normalized to the baseline (predrug) condition. Each color line represents a single culture, with the thick black line representing the mean of all cultures. ***C***, Following bicuculline, SRWB for most individual sorted units increased and were homeostatically restored over a 24 h period. Values at each time point were normalized to the baseline (predrug) condition. Each line represents a single unit from one of four cultures (units from same cultures are of the same color), with the thick black line representing the mean of all units. Inset: one (***C1***) or two (***C2***) waveforms were identified on two different channels (SD shown in shaded area).

It is possible that individual cells regulated their SRWB and/or that the number of recruited neurons that contributed to a burst was regulated. Therefore, we looked at the number of channels contributing to a burst. Following GABAergic blockade, we found that a higher proportion of the channels contributed to each burst in all but one culture (Fig. 5*B*). Before bicuculline treatment different subsets of channels contributed to any given burst, but after GABAergic blockade, the majority of the active channels contributed to most bursts. After 18 h of bicuculline treatment, the number of channels per burst recovered to predrug values (Fig. 5*B*). We then looked to identify the SRWB for single units. Different waveforms were identified on individual channels at the time the recordings were obtained based on principal component analysis of the waveform in the TDT acquisition program (Fig. 5*C1*–*C2*). We then examined channels that only contained one waveform (Fig. 5*C1*). We only accepted these individual waveforms if <1% of the interspike intervals were <2 ms (refractory period for the spike). This eliminated certain cultures entirely and the vast majority of the channels across cultures, while leaving us with 12 single-unit recordings that we could follow across conditions. We found that most of the individual units (11/12) increased to some extent following disinhibition and then returned to lower levels over the next 24 h (Fig. 5*C*). However, SRWB for individual units did not respond uniformly, consistent with the idea that there is heterogeneity in the population of recorded cells—some increased dramatically, some less so, one remained largely unchanged through the perturbation, and some (2 of 12) increased slightly but then went below 50% of their original levels. Together, these results suggested that the increase and homeostatic recovery of SRWB was occurring both at the level of individual cells and the population.

### SRWB is homeostatically restored following GABAergic blockade in the isolated chick embryo spinal cord

**Figure 6.**
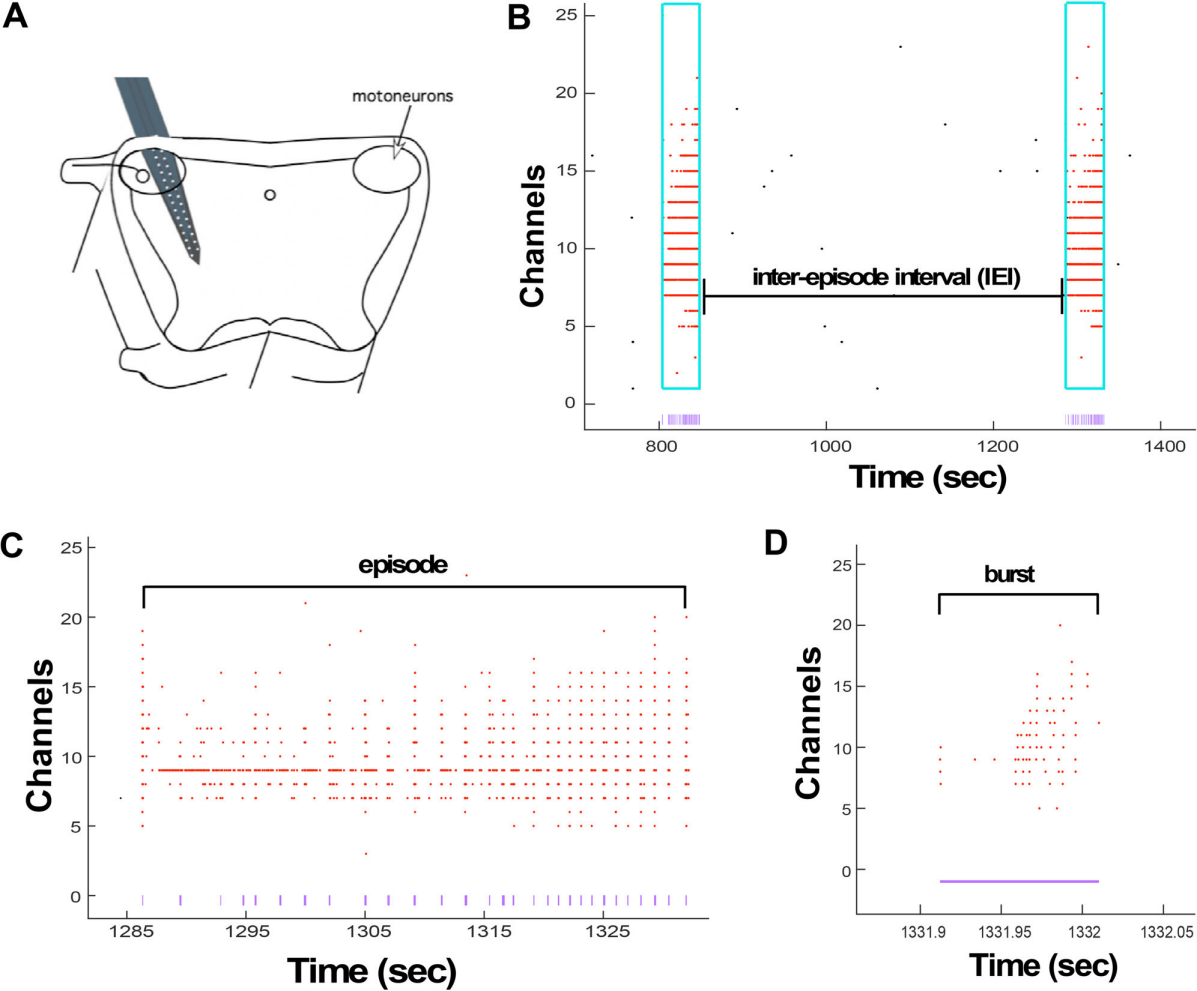
Firing rate characteristics analyzed in the isolated embryonic chick spinal cord. ***A***, Schematic of 32-channel NeuroNexus probe inserted into the spinal cord, specifically penetrating the motor column. ***B***, Raster plot of network burst and episode activity across channels on the NeuroNexus probe. Episodes caught by the custom-written Matlab program are highlighted in blue, with spikes caught in the episodes as red dots. Purple lines below the episodes represent where bursts occurred within the episode. Black dots are spikes that occurred outside of the episode. Thus, the interepisode interval spike rate is calculated by taking the spike rate outside of the episodes. ***C***, The raster is zoomed into the last episode from ***A***. The red dots again represent the spikes within the episode and the purple lines denote the bursts. From this data, we can calculate the episode spike rate, which includes all spikes within the episode, regardless of whether or not the spikes were part of a burst. ***D***, The raster is zoomed into the last burst from ***B***. We calculated the burst duration and spike rate within the burst within episodes from this data. Extended Data Figure 6-1 provides MEA recordings showing waveforms showing motoneuron antidromic activation.

The unexpected finding that SRWB was the parameter that was best homeostatically maintained in cortical cultures was clear and interesting. However, cultured cortical networks are a somewhat artificial circuit predisposed to variability due to many aspects of this system—density of plating, glial or inhibitory neuron content, and brain regions that contribute to a particular culture (somatosensory vs visual). Therefore, it was important to see if similar results might be observed in a defined class of neurons in a more intact circuit. Thus, we chose the embryonic chick spinal cord preparation, which has been shown to express several homeostatic mechanisms following both *in vivo* and *in vitro* blockade of GABAergic transmission (Wenner and Bülow, 2019; Wenner, 2020). An important difference from cortical cultures is that GABA is depolarizing and excitatory in the embryonic spinal cord (Gonzalez-Islas and Wenner, 2010). As a result, the spinal cord is highly active and recruits the majority of neurons in network-wide bursts of activity called episodes, which last for several seconds, and *in vivo*, drive embryonic movements by recruiting motoneurons (O'Donovan et al., 1998; O'Donovan, 1999). These episodes are also referred to as SNA and occur every 5–10 min; each episode is composed of several depolarizing bursts (Fig. 6; O'Donovan, 1999; Blankenship and Feller, 2010). We isolated lumbosacral spinal cords with intact ventral roots as described previously (see Materials and Methods; Gonzalez-Islas et al., 2010; Pekala and Wenner, 2022). We then drove a 32-channel NeuroNexus probe into the spinal cord (Fig. 6*A*). Antidromic stimulation of the ventral root identified which channels on the probe corresponded to motoneurons (Extended Data Fig. 6-1).

10.1523/ENEURO.0259-24.2024.f6-1 Figure 6-1 Antidromic stimulation to identify embryonic chick spinal cord motor neurons. A) Asterisks mark the component that identifies motor neurons in the raw trace. B) No motor neuron component is present in the deeper channels/interneurons. Download Fig 6-1, TIF file.

Recordings were made for ∼3–4 h, where the first 30 min of the recording provided the baseline activity. Then, excitatory GABAergic transmission was blocked with 10 µM gabazine or no antagonist was added (control). In order to better quantify spiking activity, we divided the length of the recording into 30 min time bins, and if multiple episodes were observed, the episode activity was averaged for that time bin. We analyzed overall spike rate, episode spike rate, episode duration, interepisode interval spike rate (spike rate between episodes), burst duration, and SRWB within an episode to determine which firing properties were homeostatically maintained (Fig. 6).

#### Overall spike rate/episode frequency

**Figure 7.**
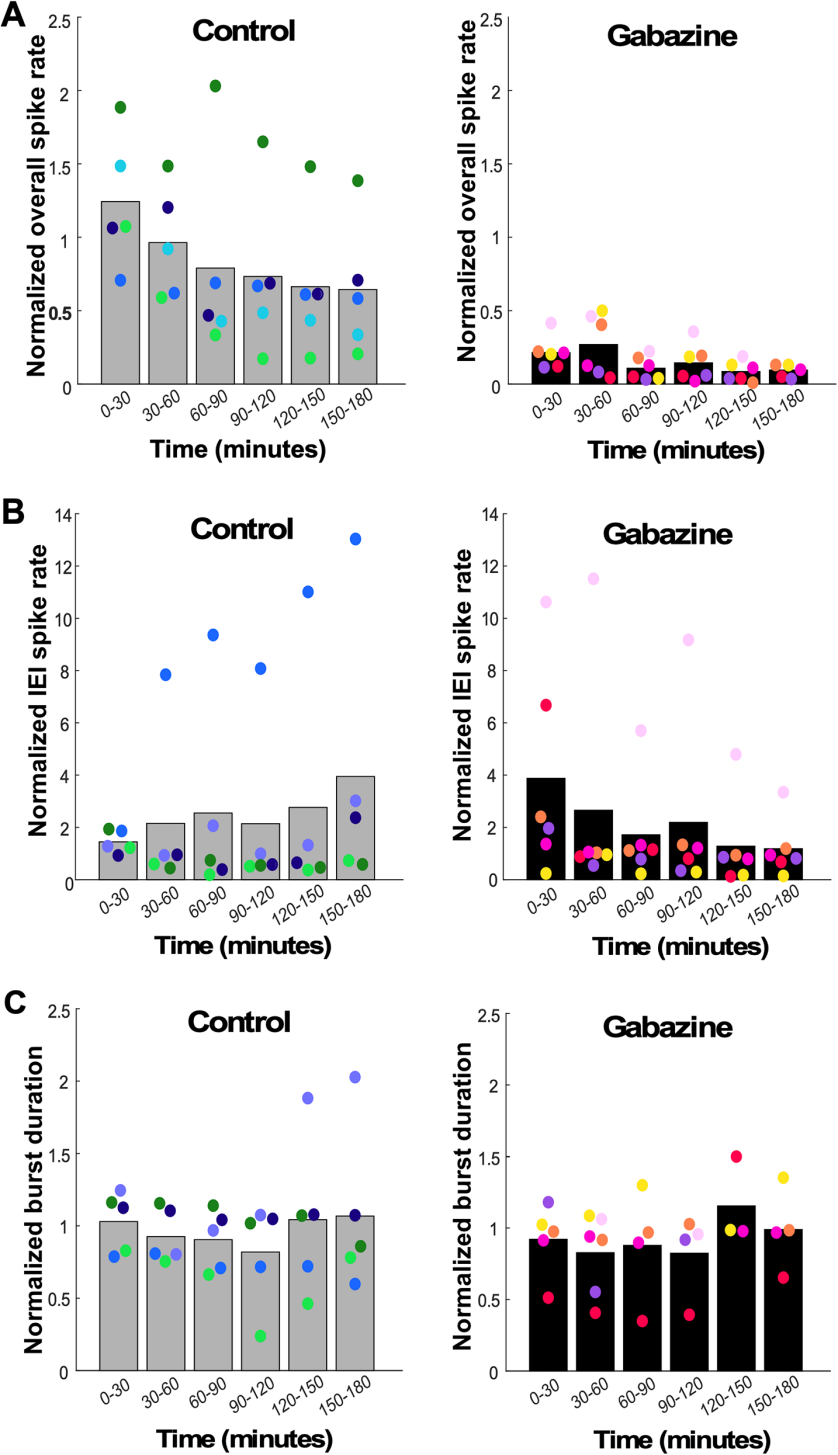
Spiking parameters measured following GABAR blockade (gabazine) in embryonic chick spinal cords. ***A***, Normalized overall spike rate, (***B***) normalized interepisode interval (IEI) spike rate, and (***C***) normalized burst duration displayed over a 3 h period for control (untreated) and gabazine-treated cords. Each color represents a single cord, with the height of the bar representing the mean of all cords. Estimation statistics comparing gabazine-treated and control cords at each of the time points establish that none were significantly different in ***B*** or ***C***. In ***A***, significant *p* values were observed for all comparisons—*p* < 0.001 for 0–30, 30–60, 60–90, and 150–180 min comparisons and *p* < 0.01 for 90–120 and 120–150 min comparisons. Extended Data Figure 7-1 provides estimation statistics for data shown in Figure 7. Extended Data Figure 7-2 shows episode frequency. Extended Data Figure 7-3 shows episode spike rate and duration.

First, we analyzed overall spike rate (Fig. 7*A*) and noticed a significant decrease in spike rate compared with the control cords within the first 30 min of gabazine addition, and this decrease persisted throughout the length of the recording (Extended Data Fig. 7-1*A*). Pregabazine values for all parameters (not normalized) are shown in Table 3. This result for the overall spike rate was not surprising as previous work has shown that most spikes occur within episodes and that blockade of a depolarizing GABAergic transmission reduced the frequency of these episodes (O'Donovan, 1999). We therefore analyzed episode frequency in these cords and found that indeed, all cords decreased the frequency of episodes following GABAergic blockade, and we did not observe signs of homeostatic recovery in the following hours (Extended Data Fig. 7-2). Because bursts are expressed in episodes, they were also reduced throughout the recording (data not shown). Since neither the overall spike rate nor the episode frequency recovered, it is clear that these features were not homeostatically maintained within the 3 h of the recording. Thus, we moved on to examine other firing rate properties to see if they were homeostatically regulated.

10.1523/ENEURO.0259-24.2024.f7-1 Figure 7-1 Estimation statistics of firing dynamic parameters following GABAR blockade (gabazine) in spinal cords for A) overall spike rate B) inter-episode interval (IEI) spike rate and C) burst duration. The mean differences at each time point were compared to control and displayed in Cumming estimation plots. Significant differences denoted by ** p < 0.01, *** p < 0.001. Upper panel shows raw data from single spinal cord recordings (filled circles), where the mean value is represented by the gap in the vertical bars and the SD is represented by the vertical bars. Lower panel shows mean differences between control and treated groups as a bootstrap sampling distribution (mean difference is represented by filled circle and the 95% CIs are depicted by vertical error bars). Download Fig 7-1, TIF file.

10.1523/ENEURO.0259-24.2024.f7-2 Figure 7-2 Episode frequency following GABAR blockade in the isolated spinal cord is not homeostatically recovered. Frequency of episodes in spinal cords is displayed as the time interval from the last episode. Each color dot represents a single spinal cord. A) Episode frequency of control cords. B) Episode frequency of gabazine-treated cords. Gabazine was added at time point 0 seconds. Download Fig 7-2, TIF file.

10.1523/ENEURO.0259-24.2024.f7-3 Figure 7-3 Episode spike rate and episode duration following GABAR blockade were variable in both control and gabazine-treated spinal cords. A) Episode spike rate displayed over a 3-hour period for control (untreated) and gabazine-treated cords. Values in each 30-minute bin are normalized to baseline condition. Each color dot represents a single spinal cord, with the height of the bar representing the mean of all cords. B) Episode duration displayed over a 3-hour period for control (untreated) and gabazine-treated cords. Values in each 30-minute bin are normalized to baseline condition. Each color dot represents a single spinal cord, with the height of the bar representing the mean of all cords. Download Fig 7-3, TIF file.

**Table 3.** Mean and standard deviation of all spiking activity features that were analyzed for spinal cord preparations

Feature	Pregabazine value
Overall spike rate	0.19 ± 0.06
Interepisode interval spike frequency	0.01 ± 0.01
Burst duration	0.09 ± 0.02
Episode spike rate	0.13 ± 0.04
Episode duration	42.74 ± 8.16
Spike rate within a burst	1.72 ± 0.71

All features are reported in Hz, except for burst duration and episode duration (seconds).

#### Interepisode interval spike rate

Next, we analyzed spiking within the interepisode interval, or the spike rate outside of episodes (Fig. 7*B*). We observed variability in both the control and gabazine-treated spinal cords, such that no significant differences were observed at any of the time points and therefore there was no homeostatic recovery of interepisode interval spike rate (Extended Data Fig. 7-1*B*).

#### Episode duration and spike rate

We next computed firing properties associated with the episode. We calculated the episode spike rate and episode duration (Extended Data Fig. 7-3). Both of these firing properties were variable in control and gabazine-treated cords, though both were clearly more variable after gabazine treatment. On average, neither property showed any significant changes following gabazine treatment. Further, any changes in individual cords that occurred following gabazine appeared to be maintained. Therefore, neither episode spike rate nor episode duration showed homeostatic regulation. These results showed that GABAergic blockade reduced the frequency of episodes, but when they occurred, the average duration and spike frequency within the episode were unaltered. However, it was clear that gabazine altered these parameters in very different ways in each individual cord (increasing in some, decreasing in others).

#### Burst duration

We also examined the duration of the bursts within the episode (Fig. 7*C*). Again, we observed no difference in the average values between gabazine-treated and control cords (Extended Data Fig. 7-1*C*). Yet, once again burst duration was highly variable in the first 90 min of GABAergic blockade. Homeostatic control of burst duration was not observed.

#### Spike rate within a burst

**Figure 8.**
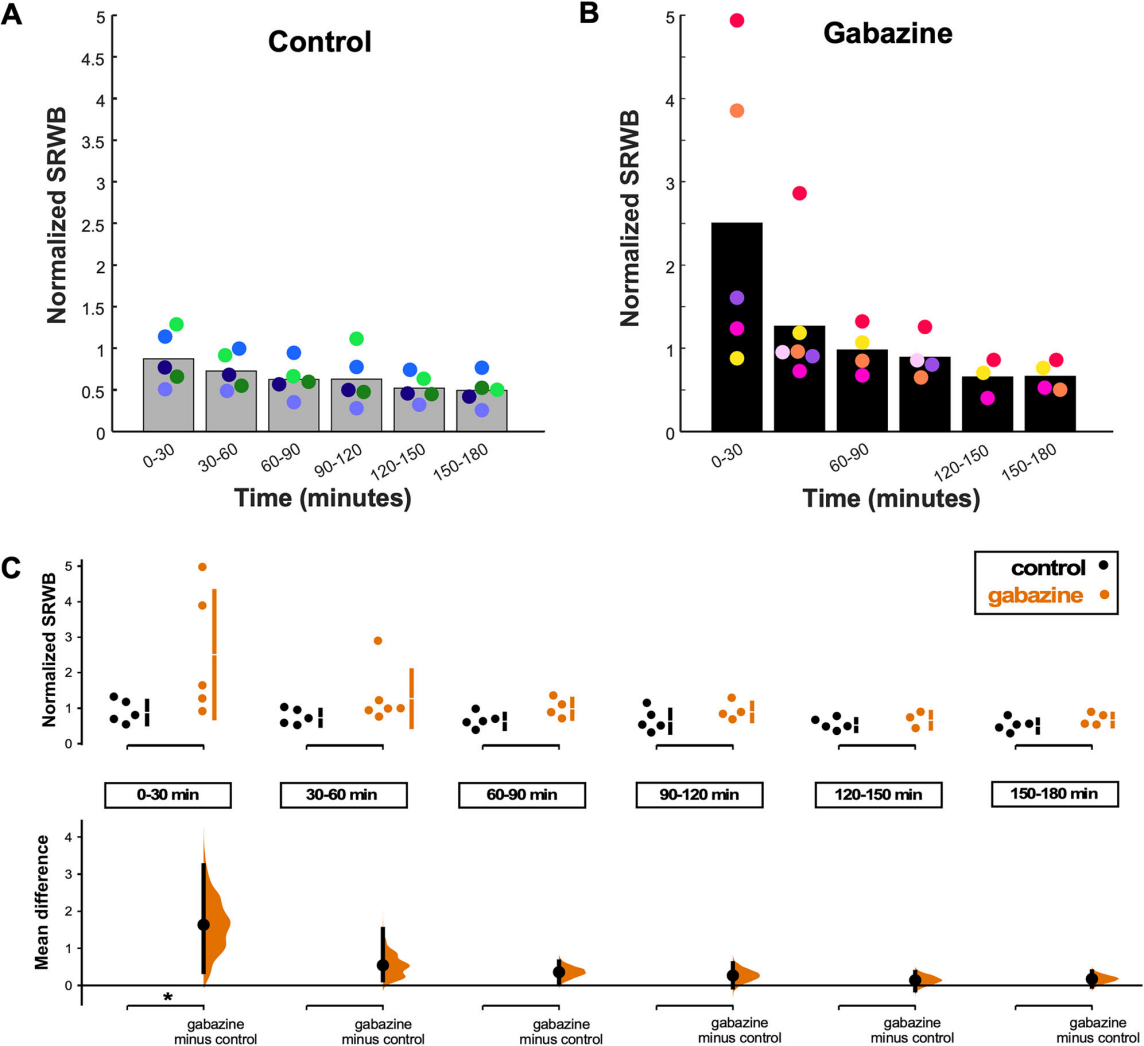
Spike rate within a burst (SRWB) within episodes following GABAR blockade in the isolated spinal cord was consistently homeostatically recovered. ***A***, ***B***, SRWB displayed over a 3 h period for (***A***) control (untreated) and (***B***) gabazine-treated cords. Values in each 30 min bin are normalized to baseline (predrug) condition. Each color represents a single spinal cord, with the height of the bar representing the mean of all cords. ***C***, SRWB is compared for control and gabazine-treated cords at 30 min intervals during the 3 h recording period. The mean differences at each time point are compared with control and displayed in Cumming estimation plots. Significant difference is denoted by *p < 0.05. The top panel shows raw data from single spinal cord recordings (filled circles), where the mean value is represented by the gap in the vertical bars and the SD is represented by the vertical bars. The bottom panel shows mean differences between control and treated groups as a bootstrap sampling distribution (mean difference is represented by filled circle and the 95% CIs are depicted by vertical error bars).

Finally, we examined SRWB, in this case, in bursts within an episode (Fig. 8*A*,*B*). We found that although the SRWB was highly variable in the first 30 min following gabazine addition, there was a significant increase in this firing property compared with the control (Fig. 8*C*). Moreover, the SRWB after the first 30 min then decreased and was similar to control cords that were not treated with gabazine. These bursts in the spinal cord drive the kicking behavior that we have observed in ovo, suggesting that the behavioral output of the system was being maintained at a specific set point. In this case, changes in SRWB would likely manifest as changes in movement amplitude or speed. Regardless, these changes in SRWB are the same as those observed in the disinhibited cortical cultures. This demonstrated that the SRWB was one of the more important firing properties because it was homeostatically regulated (see Discussion).

**Figure 9.**
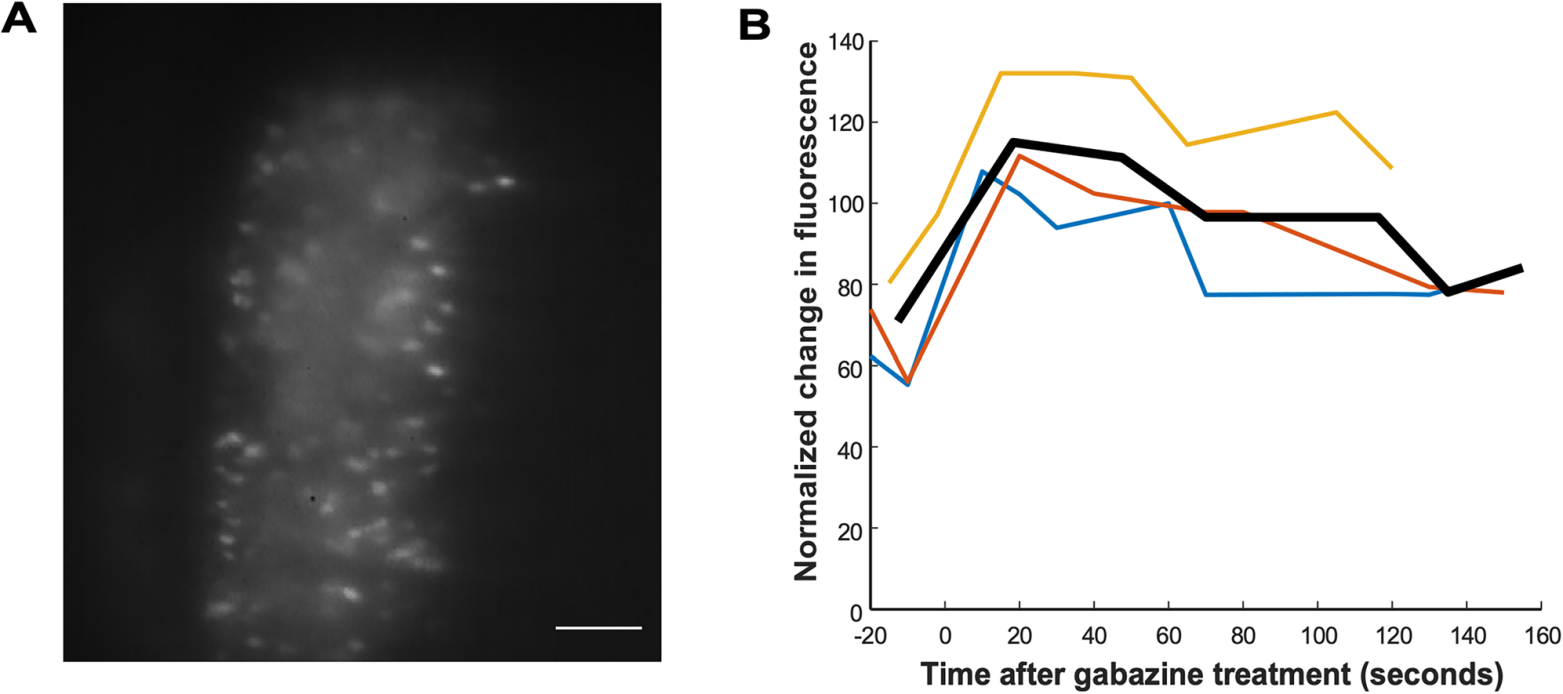
Calcium imaging of gabazine-treated embryonic chick spinal cords. ***A***, Motor neurons were labeled with Calcium Green Dextran overnight. Scale bar, 100 μm. ***B***, Results show that after gabazine addition there was an initial increase in calcium fluorescence transients associated with episodes followed by a recovery to baseline levels. Episodes were stimulated and calcium transients were recorded for three spinal cords (black line, average).

If SRWB was increased and homeostatically recovered in individual motoneurons, then we would expect to see increases in calcium entry, which are correlated with spike frequency, in individual cells. Therefore, we decided to do optical calcium imaging of individual motoneurons labeled with a calcium indicator (Fig. 9*A*). We retrogradely labeled motoneurons overnight with calcium indicator Calcium Green-1 dextran (see Materials and Methods). We then imaged the calcium transients from 10 motoneurons per cord, in three different cords (30 motoneurons total). We triggered episodes and recorded the calcium transients (% change in fluorescence) before and during 2+ h of gabazine exposure. As expected, we found that within 30 min of blocking GABAergic transmission, the calcium transient associated with an episode increased and then more slowly was reduced toward baseline values over the next 2 h (Fig. 9*B*). The result was consistent with the idea that following GABAergic blockade, individual motoneurons increased their SRWB and then homeostatically regulated this feature.

## Discussion

In this study, we found that SRWB was the spiking property that was most consistently homeostatically recovered following GABAergic blockade. We found that additional firing properties were highly variable following GABAR block, consistent with degeneracy. Further, homeostatic recovery of these other features was either weak or only occurred in some of the preparations. The homeostatic control of SRWB is logical for a circuit that must execute a specific action when the network is driven to fire during synaptic bursts.

### Variability in firing rate properties following GABAergic blockade

Following GABAR block, variability was seen across many spiking features. Individual cultures demonstrated dramatic variability in overall spike rate after bicuculline application. We might have expected an initial increase in overall spike rate since the system was disinhibited. On average, there was a slight increase in the first hours (although it did not reach significance; Extended Data Fig. 2-1*A*). This result was largely consistent with similar studies in culture (Turrigiano et al., 1998; Corner et al., 2002). However, when assessing individual cultures, we saw dramatic variability in the response to disinhibition as some increased 3–4-fold, while others nearly stopped firing. Why would disinhibition cause a reduction in overall spiking? One possibility is that it led to a depolarizing block, inactivating voltage-gated sodium channels (Bianchi et al., 2012). While the overall spike rate in cultures was highly variable following GABAergic blockade, this feature was consistently lower than control levels in the isolated spinal cord. This was due to the fact that such a perturbation reduced episode frequency, where most spikes occur. In the embryonic chick spinal cord, this makes sense because gabazine blocks a depolarizing excitatory current that is important in episode generation (Chub and O'Donovan, 1998).

Overall spike rate was highly variable even though SRWB consistently increased and then homeostatically recovered. One might have expected that overall spike rate would be impacted by SRWB, but this did not appear to be the case, as some cultures decreased overall spike rate at the same time SRWB increased, and in all spinal cord preparations overall spike rate decreased dramatically at the same time SRWB increased. This occurred because overall spike rate was not only influenced by SRWB, but also burst frequency, burst duration, and IBI spiking. For instance, in the spinal cord, overall spike rate decreased due to a reduction in the episode frequency and therefore burst frequency. Interestingly, the interaction of SRWB with other activity features was itself variable. The interaction of these features can be seen by comparing different cultures. For example, in the culture identified by the red line in Figures 2 and 3, SRWB goes up for 9 h and then recovers (Fig. 3*B*), which is similar to overall spike frequency (Fig. 2*A*), but different than burst frequency (goes down and stays there; Fig. 2*B*) or burst duration (goes up and stays there; Fig. 2*D*). On the other hand, the culture identified by the purple line has a typical increase and recovery of SRWB within ∼3 h, which is different than overall spike rate or burst duration (goes down and stays there) or burst frequency (largely unchanged).

Why does such variability exist? This is consistent with the initial hypothesis that there is significant variability in synaptic strengths and voltage-gated conductances within neurons in circuits expressing similar activity patterns (degeneracy; Liu et al., 1998; Prinz et al., 2004; Hamood and Marder, 2014; Goaillard and Marder, 2021; Yang and Prescott, 2023). This variability is thought to be driven by homeostatic plasticity goals (e.g., achieving proper activity patterns), but different preparations use different strategies to accomplish this. In the context of development, these results are particularly profound because the many challenges during development are even more consequential and will therefore strongly shape the strategies used to accomplish homeostasis. Thus, the response to a perturbation will depend on the strategies chosen by that particular network. Therefore, it may not be surprising that networks with similar behaviors respond so differently to distinct perturbations. For instance, a network that developed using a strategy more strongly dependent on GABAergic synaptic strength would likely be more affected by GABAergic blockade. The observations that different constellations of ionic conductances and synaptic strengths can produce similar activity patterns and that perturbations can uncover such variability have been well documented in invertebrate systems (Prinz et al., 2004; Goaillard and Marder, 2021). However, similar experiments have been carried out far less in vertebrate preparations, and our results appear to support the concepts initially recognized in invertebrates. Finally, this variability is obvious, but the recognition of this is often lost when simply looking at average values, as suggested several years ago (Golowasch et al., 2002).

### Homeostasis of SRWB in both preparations

In both the isolated spinal cord and cortical cultures, GABAergic blockade induced an increase in SRWB that displayed far less variability than other activity features—all cultures and four of five spinal preparations increased SRWB, which then recovered. The increase and homeostatic recovery of SRWB appear to be due to changes that are occurring at the individual cellular level as these changes were observed in single-unit recordings in culture and through calcium imaging of individual motoneurons in the spinal cord (Figs. 5, 9). Such changes are likely to be driven, at least in part, by a shift in the driving force for synaptic currents underlying bursts. In control spinal cords, the currents associated with SNA are predominantly glutamatergic and GABAergic. Previous work has demonstrated that the reversal potential for SNA in E10 chick spinal neurons was approximately −20 mV (GABAergic and glutamatergic), close to the GABA reversal potential of −30 mV, and this then shifted to ∼0 mV after the addition of bicuculline (now only glutamatergic; Chub and O'Donovan, 1998). The GABA reversal potential is close to the membrane potential during a burst and so it can actually shunt action potentials. Moreover, when GABA receptors are blocked, fewer chloride channels would be open, thus increasing the input resistance. In the chick embryo, it has been shown that puffing a GABAR antagonist onto the cord during SNA can increase burst discharge acutely, presumably by shutting off what can be a shunting conductance (Sernagor et al., 1995). In cultured cortical neurons, we would expect a similar mechanism underlying the increased SRWB following GABA receptor blockade, as input resistance should also increase and we are removing a synaptic current that is hyperpolarizing. The increase in SRWB following GABAergic blockade appears to be similar to that reported previously (Corner and Ramakers, 1992; Corner et al., 2002; Weihberger et al., 2013). The consistency across preparations of increased SRWB is likely due to the twofold nature of a stronger inward current in a tighter cell (higher input resistance). Such a mechanism could also underlie the recruitment of more neurons into each burst as more of the population is brought to threshold.

Since we saw the recovery of SRWB while GABAR blockade was still in effect, there must be a mechanism to bring this firing rate back to control levels that is independent of GABAR activation. We hypothesize that this effect could be due, in part, to a depolarization of the resting membrane potential (Gonzalez-Islas et al., 2020). Previous work has shown that the resting membrane potential was depolarized ∼10 mV in motoneurons 1–2 h after GABAergic blockade. While this could suggest the cells are more excitable and could increase firing rate, it is possible that in combination with a more depolarized reversal potential for SNA, the cells could enter depolarizing block, thus inactivating voltage-gated sodium channels (Chub and O'Donovan, 1998). In addition, or alternatively, it is possible that rapid activity-dependent changes in intrinsic cellular excitability, such as K^+^ channel current densities, mediate the recovery. Following reductions in voltage-gated Na^+^ channel conductances, acutely dissociated Purkinje cells maintained spikes within a burst through rapid reductions in K^+^ channel currents (Swensen and Bean, 2005). In the stomatogastric ganglia, it was shown that depolarization of an all-inhibitory synaptic network reduced spikes within a burst, which then began to recover within the first hour by hyperpolarizing the action potential threshold, potentially through reduced K^+^ channel conductance (He et al., 2020).

It remains unclear how the increase in SRWB is sensed and then triggers whatever homeostatic mechanism that restores this feature. One possibility is that the increased firing rate leads to an increased calcium transient (Fig. 9) that could trigger calcium signaling cascades that lead to the appropriate homeostatic mechanisms that restore SRWB, thus avoiding cellular hyperexcitability that could be deleterious for the cell. Regardless of the mechanism, homeostatic control suggests an elevated importance of this feature as it requires a sensor of the feature and the metabolic cost to regulate it. Further supporting the importance of the finding is the fact that we see this homeostatic regulation of SRWB in two very different systems—two different cell types, species, temperatures, GABAR antagonists, and developmental stages.

Previous work has often been ambiguous about the exact variable that is homeostatically regulated, but in general, studies have focused on the recovery of overall spike rate after a perturbation. However, our current study suggests that SRWB is one important variable that is tightly homeostatically controlled in both the in vitro cortical cultures and ex vivo embryonic spinal cords. Although we did not see evidence of a full recovery in the overall spike rate in the embryonic chick spinal preparation, embryonic movements in ovo did recover after 12 h (Wilhelm et al., 2009). Thus, some homeostatic mechanisms must exist in the fully intact living embryo for this recovery. This may be due to the fact that we only looked at the first 3+ h of recovery or there may be compensations in the periphery, descending input, or neuromodulators that are not recapitulated in the isolated ex vivo cord. Regardless, SRWB initially increases and then robustly returns to baseline levels in these preparations. Therefore, SRWB represents a critical firing rate property that is homeostatically maintained in a reliable manner but is rarely considered in homeostatic studies. The bursts in culture are representative of activity that the network generates, while the bursts in the spinal cord drive kicking behavior that we have previously observed in ovo. In both preparations, the bursts are the synaptic outcome of the system, and the spike rate within these bursts is the feature that is consistently homeostatically regulated following this perturbation.

## Synthesis

Reviewing Editor: Tod E Kippin, University of California Berkeley Decisions are customarily a result of the Reviewing Editor and the peer reviewers coming together and discussing their recommendations until a consensus is reached. When revisions are invited, a fact-based synthesis statement explaining their decision and outlining what is needed to prepare a revision will be listed below. The following reviewer(s) agreed to reveal their identity: Andrew Alexander.

I have received two independent reviews of your manuscript both reviewers felt the study was interesting, generally well-conducted, and timely. The reviewers did have a few minor concerns which need to addressed before a final decision can be reached on suitability for publication. I am, accordingly, recommending that you revise your manuscript. I am happy to be of assistance during the revision process if anything is unclear.

Reviewer#1

Here, the authors look at homeostatic plasticity in neuronal cultures and embryonic spinal cord following blockade of GABA transmission. They observe that GABA antagonism causes highly variable changes to numerous activation properties that are not homeostaticaly regulated. However, in both preps, GABAergic inhibition resulted in an increase in spike rate within bursts (SRWB) that returned to baseline levels after extended temporal durations. The authors conclude that SRWB must be an important firing property because it is homeostatically regulated following perturbation. I have several minor comments that should be addressed in a revised manuscript.

Many of the firing rate properties are treated as independent but are likely conflated. For instance, overall firing rate should be (at least minimally) impacted by the spike rate within bursts. The authors primarily isolate particular epochs for independent analysis but I'm curious what the correlation between these different variables is. An analysis or discussion of the influence of each variable on other parameters is warranted. It is not immediately clear to me why one property would be homeostatically regulated independently of others.

Why was 20uM of bicuculline used. Did the authors consider trying multiple concentrations to see if there was a particular dose level that reduced the variability in neuronal culture? Also, why is a different GABAergic antagonist used in the spinal cord?

I don't understand why the SRWB increases in the spinal cord prep if GABA is excitatory. Shouldn't blockade reduce the SRWB and exhibit a pattern opposite of the culture prep?

Figure 4 - The homeostatic index doesn't look like it is significantly different between measurements, did the authors test this?

"These studies demonstrate a critical importance of this firing rate property where the network is synaptically driven to "behave"."

-I don't understand this, please elaborate. How does SWRB relate to the network returning to homeostasis?

How does SWRB relate to the other metrics? If it is returning to baseline how does that also not affect spike rate? How correlated are these metrics within culture?

"Before bicuculline treatment different subsets of channels contributed to any given burst, but after GABAergic blockade, the majority of the active channels contributed to most burst."

-Can you clarify how many channels were active at a given time and number involved in a burst in control cultures? Otherwise this isn't really supported by what is presented.

Are the single unit recordings in 5B taken from multiple cultures?

L433 "This demonstrated that the SWRB was one of the more important firing properties because it was homeostatically regulated." - I don't understand this, please clarify.

L439 - Why only 10 motorneruons per cord? It looks like there are more than 10 cells visible in 9A, why not analyze them all?

Reviewer#2

The authors investigate response profiles of multiple preparations to GABAergic blockade and identify a key variable that is most consistently changed. One of the most interesting findings is that many of the measured variables exhibit a very high degree of variance, whilst SRWB is most consistenetly changed. The experimental design is appropos and I appreciated the evaluation across multiple model systems. My one suggestion would be that perhaps the variance in the cultured neurons has to do with the potential brain regional source of said neurons. I hold to the opinion that visual cortex is different than somatosensory cortex (for example) and that this may be driving some variance.

minor point

-(unknown sex) in abstract has no context and should be reworded.

## Author Response

We thank the editor and reviewers for their positive responses and suggestions to improve the manuscript. Our responses to the reviewers' suggestions are given below in blue.

Reviewer#1 Many of the firing rate properties are treated as independent but are likely conflated. For instance, overall firing rate should be (at least minimally) impacted by the spike rate within bursts. The authors primarily isolate particular epochs for independent analysis but I'm curious what the correlation between these different variables is. An analysis or discussion of the influence of each variable on other parameters is warranted. It is not immediately clear to me why one property would be homeostatically regulated independently of others.

The firing properties don't have to be dependent on one another and in many cases are not correlated. For instance, in the spinal cord preparations, we see that overall firing rate drops with a GABAergic blockade, but the SRWB actually increases immediately following blockade before homeostatically returning to baseline levels, even though the overall firing rate never recovers. Overall firing rate is dependent on SRWB, but also burst frequency/burst duration/inter-episode interval. We now elaborate on this in the discussion (see lines 479-494). We do discuss the idea that SRWB is homeostatically regulated because this is the output of the network when it is behaving/active. Further arguments are made below (lines 335-337, 460-461, 580-584).

Why was 20uM of bicuculline used. Did the authors consider trying multiple concentrations to see if there was a particular dose level that reduced the variability in neuronal culture? Also, why is a different GABAergic antagonist used in the spinal cord? We did not test multiple concentrations of bicuculline, but 20uM was a typical concentration that we have used in the past and within the range used in literature. We also did use a relatively low concentration of gabazine for SRWB in cultures as well (Figure 3-2). This lesser blockade of GABARs produced a very similar response to SRWB, and because it has not been reported to block K+ channels, as bicuculline has been, we continued with this blocker for the chick embryo. This is discussed in lines 313-316.

I don't understand why the SRWB increases in the spinal cord prep if GABA is excitatory. Shouldn't blockade reduce the SRWB and exhibit a pattern opposite of the culture prep? This is addressed in the Discussion of the paper, and we have tried to clarify this. Briefly, although GABA is excitatory in the chick embryo, the reversal potential is around -30mV, which is near the membrane potential during a burst, so it would actually resist the further depolarization and thus recruitment of voltage gated Na+ channels and because the GABAR/chloride channels are open they will make the cell leakier and therefore harder to fire an action potential. Once GABAergic currents are removed/blocked, the cell is tighter and the reversal potential for the SNA is depolarized from -20 to 0 mV - both would make the cell more likely to spike. This is discussed in lines 522-541.

Figure 4 - The homeostatic index doesn't look like it is significantly different between measurements, did the authors test this? The averages of the homeostatic index are not significantly different from each other for any of the features; however, we are highlighting the consistency of the preparations in returning to control values. Therefore, we are more interested in the mean % recovery and the variability in this measure across preparations - coefficient of variation (CV). Therefore, we have now added a table of CV and mean recovery value. It is clear that SRWB has the lowest CV (0.19) and returns closest to 100% (90.32%). Here is a table (new Table 2) of the CV and mean recovery for all the firing rate properties, which we have been included in the resubmission (lines 333-335): "These studies demonstrate a critical importance of this firing rate property where the network is synaptically driven to "behave"." -I don't understand this, please elaborate. How does SWRB relate to the network returning to homeostasis? In the cortical cultures, SRWB is homeostatically recovered best, and our point here was that this feature is the critical feature that occurs when the network is behaving or in other words synaptically-driven to be active, rather than maintaining the number of times it is active or total number of spikes. This is addressed in lines 335-337.

How does SWRB relate to the other metrics? If it is returning to baseline how does that also not affect spike rate? How correlated are these metrics within culture? These questions are similar to the earlier comment about firing properties being potentially dependent on one another, but it turns out these parameters are often not correlated in a consistent way. While each one of these parameters can influence the others, each culture appears to get it done in a slightly different way and it is often not straight forward. For instance, an increase in SRWB could increase overall firing rate, but not if burst frequency goes down. One can see this by comparing the different cultures in figures 2 and 3 (the line colors can be used to identify certain cultures - e.g. the red lines are the same culture in all of bicuculline-treated cultures in Figures 2 and 3). We have added this to the discussion (lines 479-494). "Before bicuculline treatment different subsets of channels contributed to any given burst, but after GABAergic blockade, the majority of the active channels contributed to most burst." -Can you clarify how many channels were active at a given time and number involved in a burst in control cultures? Otherwise this isn't really supported by what is presented.

We have now added a plot to Figure 5 that displays the number of channels involved in a burst for the control cortical cultures (Figure 5A). The figure shows that the number of channels remains consistent in the control cultures, and Figure 5B shows that the number of channels involved in a burst following bicuculline treatment increases and then homeostatically recovers to the pre-drug condition. In addition, we have added the number of channels involved in the burst at baseline, before bicuculline was added to cultures in Table 1 (mean and standard deviation). The mean and standard deviation for the control cultures was 15.54 + 4.07 number of channels.

Are the single unit recordings in 5B taken from multiple cultures? Yes, the single unit recordings are taken from 4 different cultures. This is mentioned in the figure 5 legend "Each line represents a single unit from one of four cultures (units from same cultures are of the same color), with the thick black line representing the mean of all units." L433 "This demonstrated that the SWRB was one of the more important firing properties because it was homeostatically regulated." - I don't understand this, please clarify.

In the discussion, we elaborate on this further (lines 562-567)- "Regardless of the mechanism, homeostatic control suggests an elevated importance of this feature as it requires a sensor of the feature and the metabolic cost to regulate it. Further supporting the importance of the finding is the fact that we see this homeostatic regulation of SRWB in 2 very different systems - 2 different cell types, species, temperatures, GABAR antagonists, and developmental stages." The fact that the cell regulates this one property more than others suggests that energy is spent to ensure that SRWB is maintained. We are not saying that this is the only feature that is regulated in a homeostatic fashion, but that of the features we have examined, SRWB is the best homeostatically regulated feature in two different systems.

L439 - Why only 10 motoneurons per cord? It looks like there are more than 10 cells visible in 9A, why not analyze them all? There are more than 10 motoneurons per cord, but in order to avoid biassing our data to one cord (the one with the most motoneurons), we limited our analysis to 10 motoneurons per cord for each cord.

Reviewer#2 My one suggestion would be that perhaps the variance in the cultured neurons has to do with the potential brain regional source of said neurons. I hold to the opinion that visual cortex is different than somatosensory cortex (for example) and that this may be driving some variance.

We agree that different brain region sources could account for some of the variance seen in the cortical cultures, and we have now included this in the results section. We actually think this strengthens the importance of SRWB in that its recovery was seen in all of the cultures, despite the fact that cultures can be quite variable from one to the next. We added the following text (lines 366-369): "However, cultured cortical networks are a somewhat artificial circuit predisposed to variability due to many aspects of this system - density of plating, glial or inhibitory neuron content, cortical regions that contribute to a particular culture (somatosensory vs visual)." minor point -(unknown sex) in abstract has no context and should be reworded.

As per the eNeuro manuscript submission guidelines, abstract must contain "methodology (including the species and sex studied)." However, we have provided context now in the abstract (line 6).
