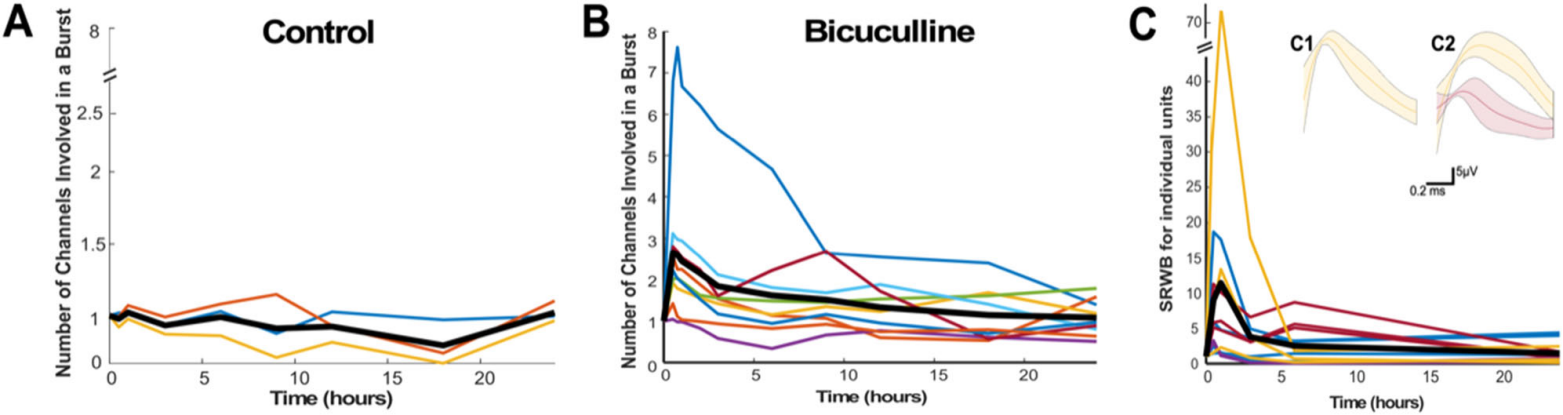# Erratum: Lakhani et al., “Homeostatic Regulation of Spike Rate within Bursts in Two Distinct Preparations”

**DOI:** 10.1523/ENEURO.0467-24.2024

**Published:** 2024-11-12

In the article “Homeostatic Regulation of Spike Rate within Bursts in Two Distinct Preparations,” by Alishah Lakhani, Carlos Gonzalez-Islas, Zahraa Sabra, Nicholas Au Yong, and Peter Wenner, which was published online on August 19, 2024, the authors wish to change the orientation of Figure 5 so that it appears horizontally rather than vertically. This correction does not affect the conclusions of the paper, and the figure has been updated online. The new version of the figure is displayed below.

**Figure 5.**