# Supplementary figures and images for: Homeostatic Regulation of Spike Rate within Bursts in Two Distinct Preparations
Source: eNeuro. 2024 Sep 3;11(9):ENEURO.0259-24.2024. doi: 10.1523/ENEURO.0259-24.2024 (PMC11391507; doi:10.1523/ENEURO.0259-24.2024)

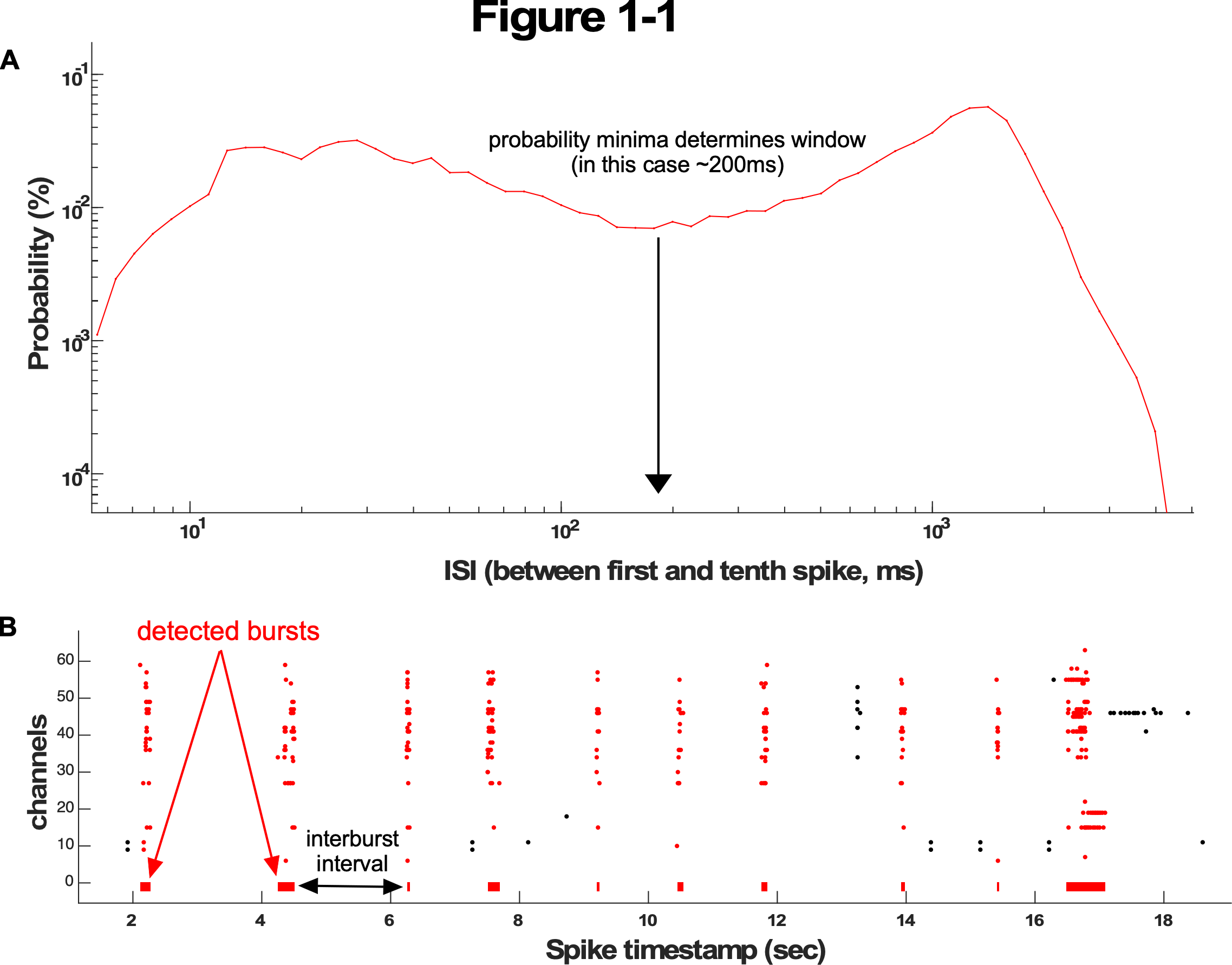

Supplement: Figure 1-1 — Program that detects and analyzes bursts. A) Probability distribution of ISI durations between 10 consecutive spikes (n to n + 9) largest dip in the distribution (200 ms) is used to detect bursts in the burst detection subroutine of the program. B) Bust detection program identifying bursts that contain at least 10 spikes in 200 ms. Burst duration is shown in red line below raster plot. Burst spikes are red and spikes in the inter-burst interval are black. Download Fig 1-1, TIF file. [file eneuro-11-ENEURO.0259-24.2024-s001.tif]

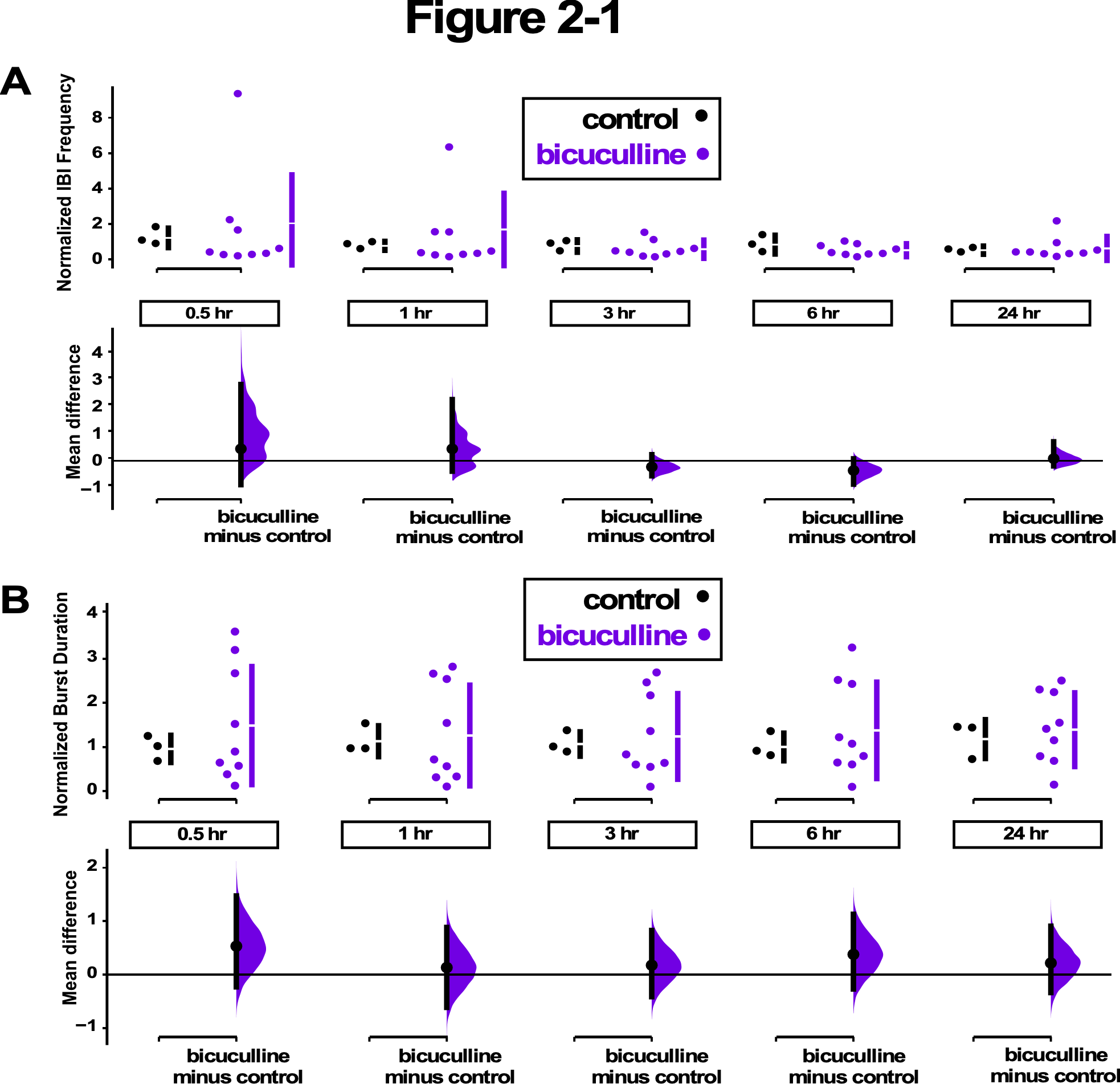

Supplement: Figure 2-1 — Estimation statistics of burst dynamic parameters from bicuculline-treated cultures for A) overall spike rate B) burst frequency C) inter-burst interval (IBI) spike rate and D) burst duration. The mean differences at each time point are compared to control and displayed in Cumming estimation plots. Upper panel shows raw data from recordings of individual cultures (filled circles), where the mean value is represented by the gap in the vertical bars and the SD is represented by the vertical bars. Lower panel shows mean differences between control and treated groups as a bootstrap sampling distribution (mean difference is represented by filled circle and the 95% CIs are depicted by vertical error bars). Download Fig 2-1, TIF file. [file eneuro-11-ENEURO.0259-24.2024-s002.tif]

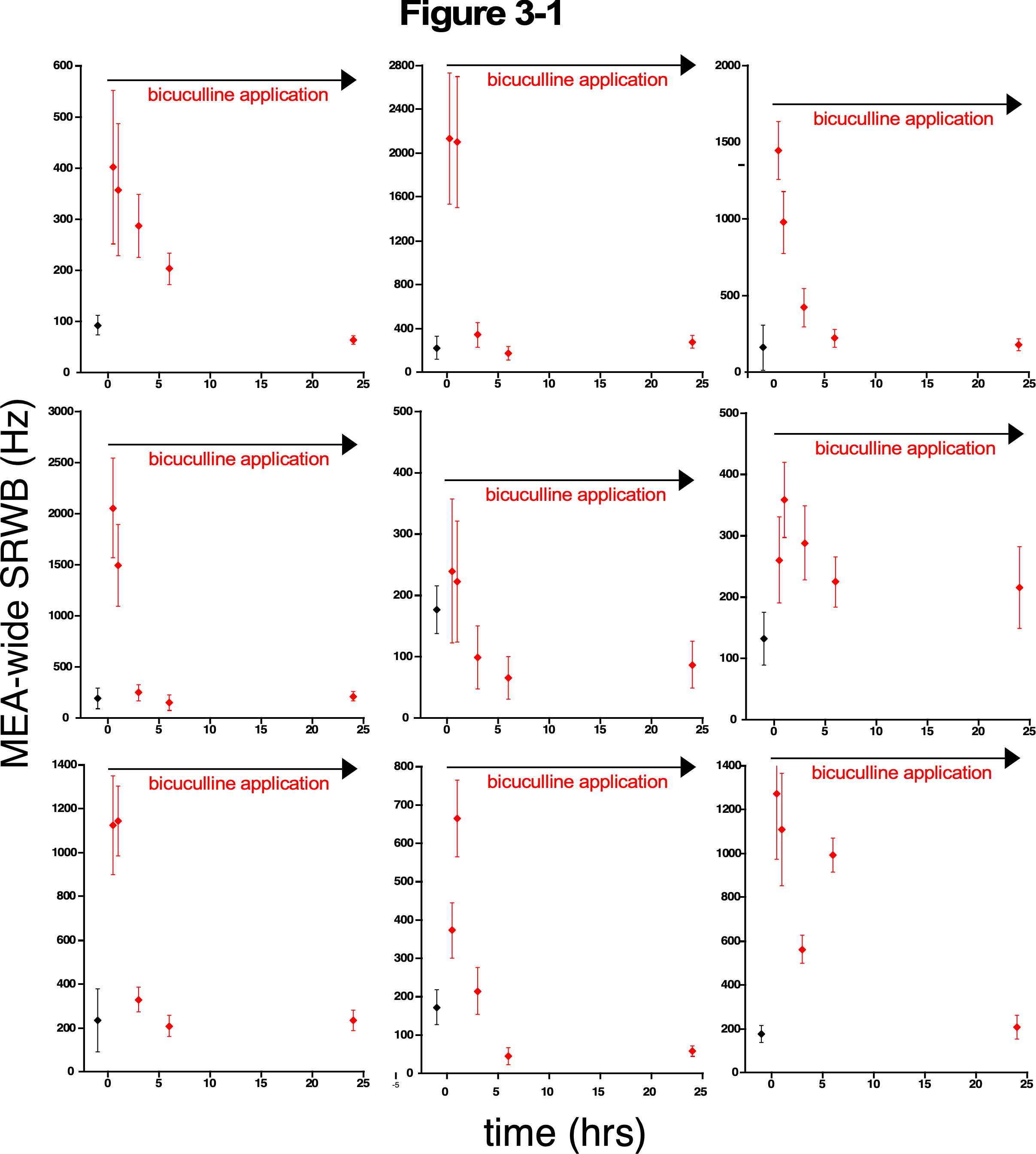

Supplement: Figure 3-1 — Nine different cortical cultures show that addition of bicuculline triggers an increase in SRWB, which then is homeostatically returned to baseline levels. SRWB is calculated as the average SRWB across individual bursts and standard deviation of SRWB is shown as error bars. Data is not normalized and therefore represents the MEA-wide spike rate within a burst of each culture before (black) and after bicuculline (red). Download Fig 3-1, TIF file. [file eneuro-11-ENEURO.0259-24.2024-s003.tif]

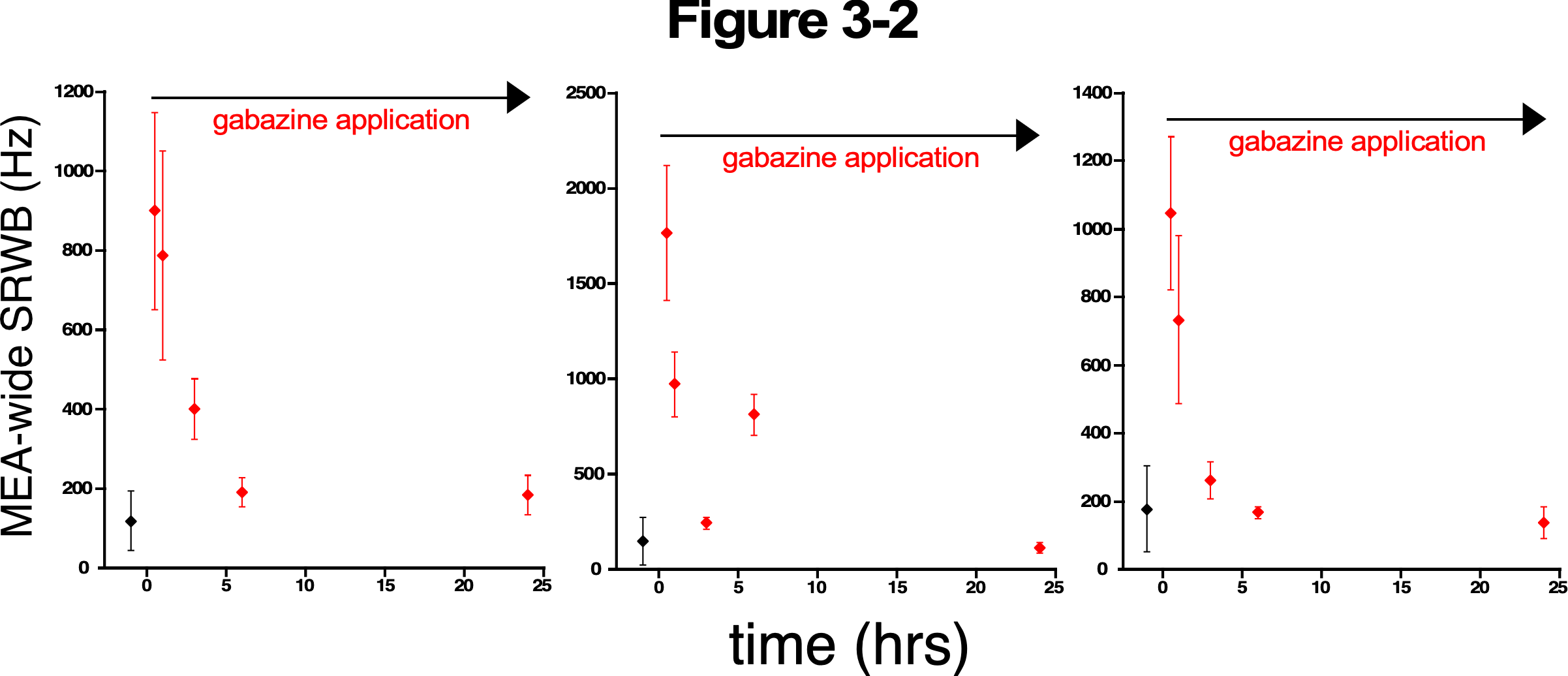

Supplement: Figure 3-2 — Three different cortical cultures show that addition of gabazine triggers an increase in SRWB, which then is homeostatically returned to baseline levels. SRWB is calculated as the average SRWB across individual bursts and standard deviation of SRWB is shown as error bars. Data was not normalized and therefore represents the MEA-wide spike rate within a burst for each culture before (black) and after gabazine (red). Download Fig 3-2, TIF file. [file eneuro-11-ENEURO.0259-24.2024-s004.tif]

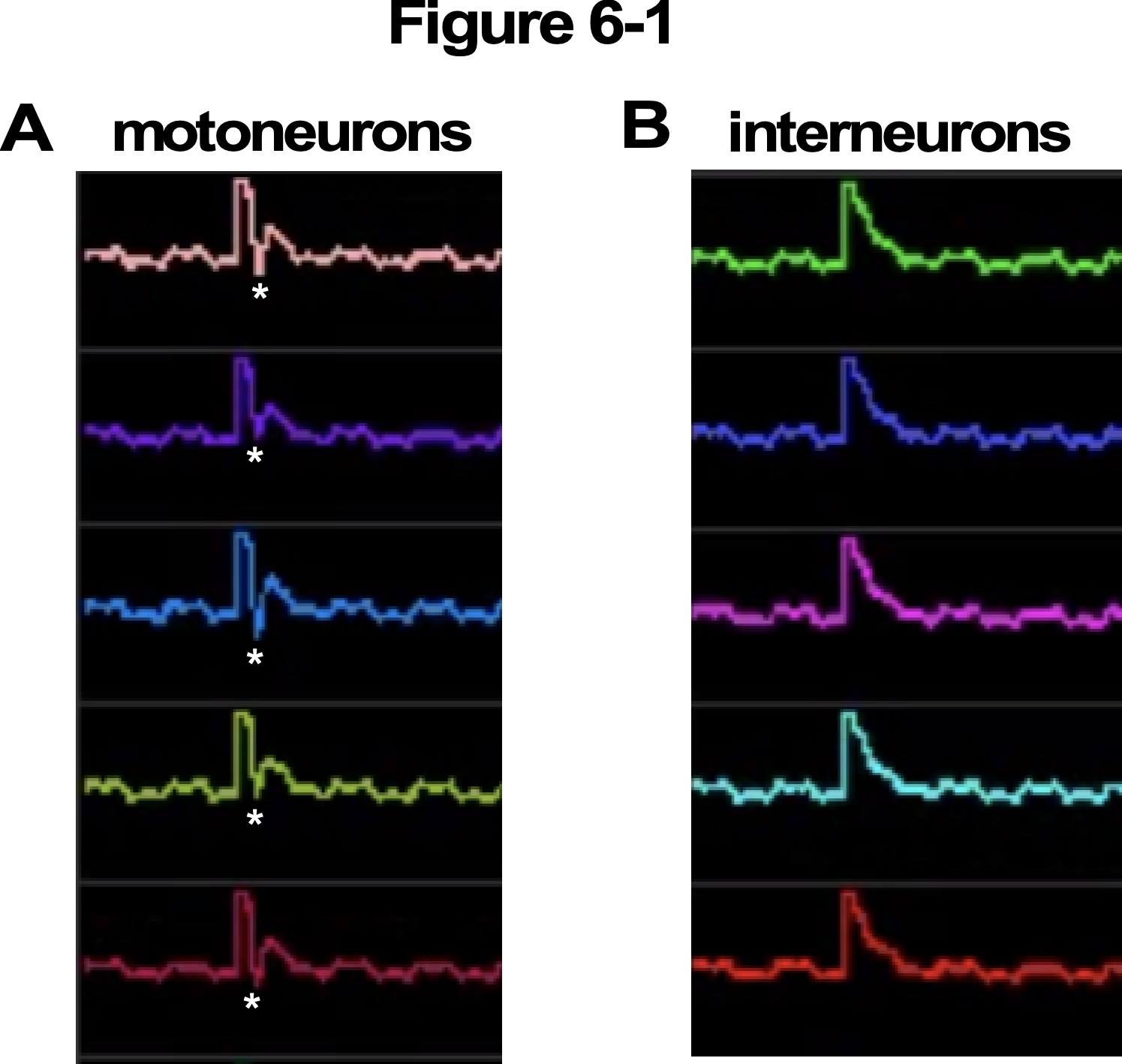

Supplement: Figure 6-1 — Antidromic stimulation to identify embryonic chick spinal cord motor neurons. A) Asterisks mark the component that identifies motor neurons in the raw trace. B) No motor neuron component is present in the deeper channels/interneurons. Download Fig 6-1, TIF file. [file eneuro-11-ENEURO.0259-24.2024-s005.tif]

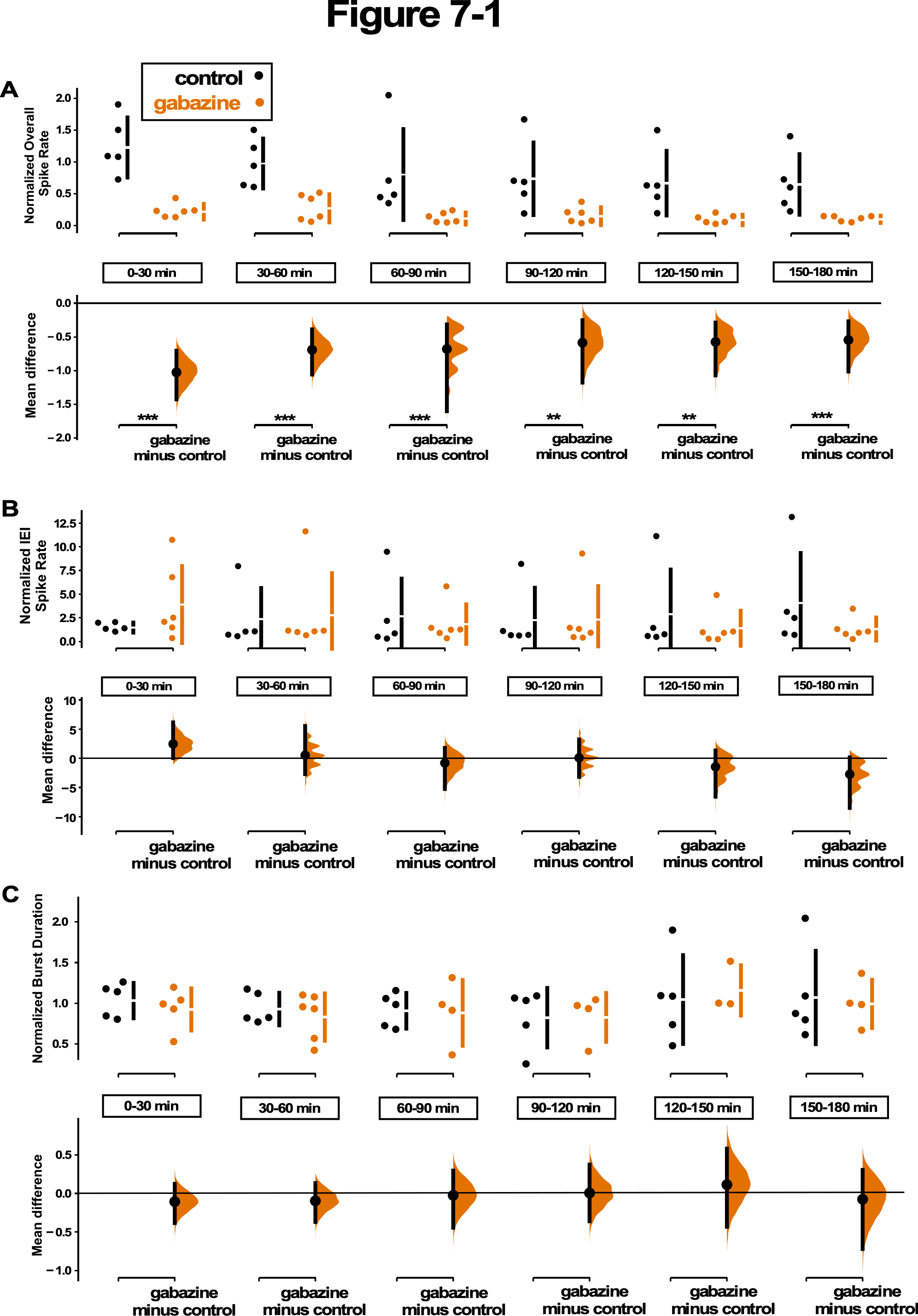

Supplement: Figure 7-1 — Estimation statistics of firing dynamic parameters following GABAR blockade (gabazine) in spinal cords for A) overall spike rate B) inter-episode interval (IEI) spike rate and C) burst duration. The mean differences at each time point were compared to control and displayed in Cumming estimation plots. Significant differences denoted by ** p < 0.01, *** p < 0.001. Upper panel shows raw data from single spinal cord recordings (filled circles), where the mean value is represented by the gap in the vertical bars and the SD is represented by the vertical bars. Lower panel shows mean differences between control and treated groups as a bootstrap sampling distribution (mean difference is represented by filled circle and the 95% CIs are depicted by vertical error bars). Download Fig 7-1, TIF file. [file eneuro-11-ENEURO.0259-24.2024-s006.tif]

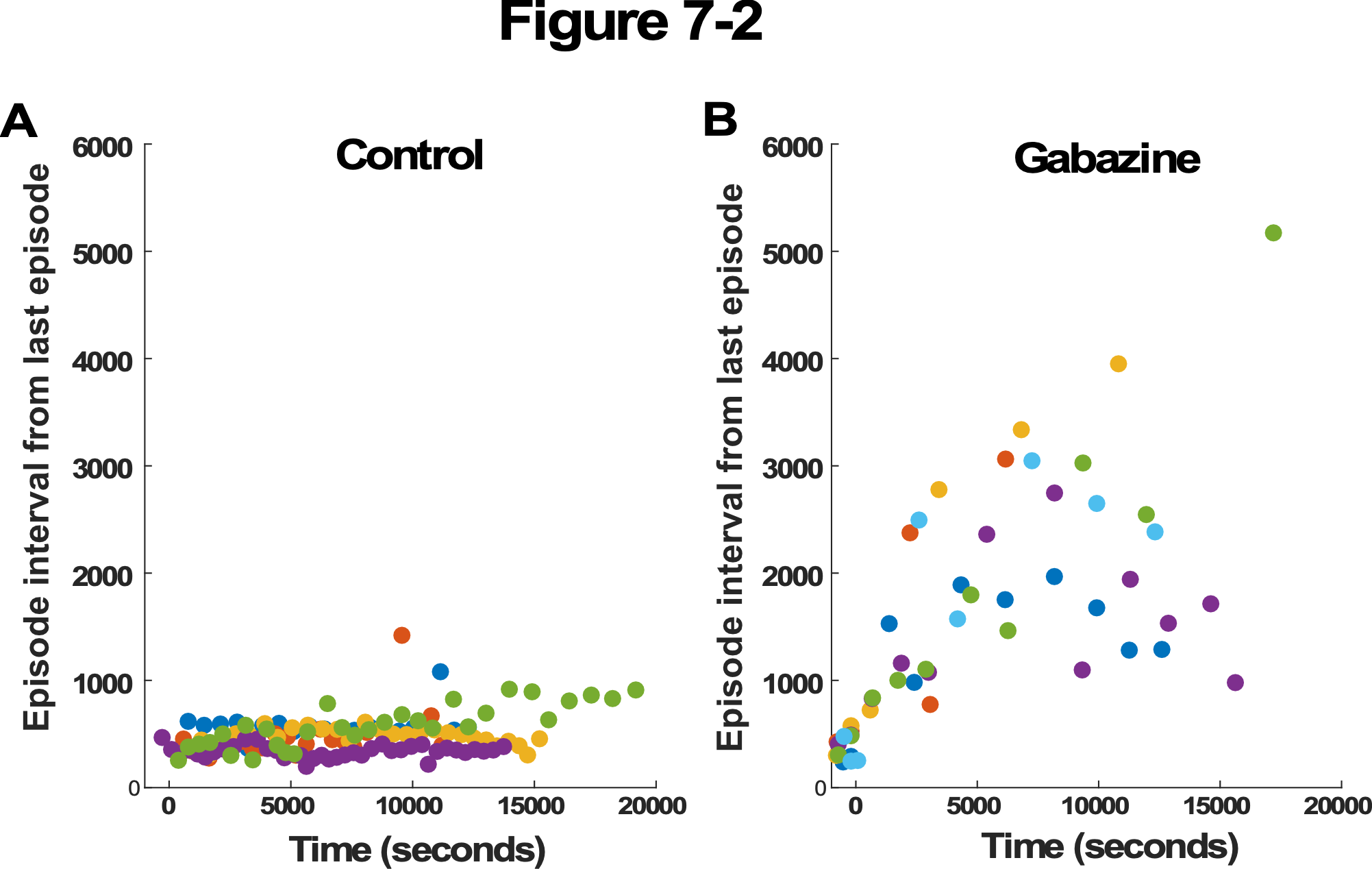

Supplement: Figure 7-2 — Episode frequency following GABAR blockade in the isolated spinal cord is not homeostatically recovered. Frequency of episodes in spinal cords is displayed as the time interval from the last episode. Each color dot represents a single spinal cord. A) Episode frequency of control cords. B) Episode frequency of gabazine-treated cords. Gabazine was added at time point 0 seconds. Download Fig 7-2, TIF file. [file eneuro-11-ENEURO.0259-24.2024-s007.tif]

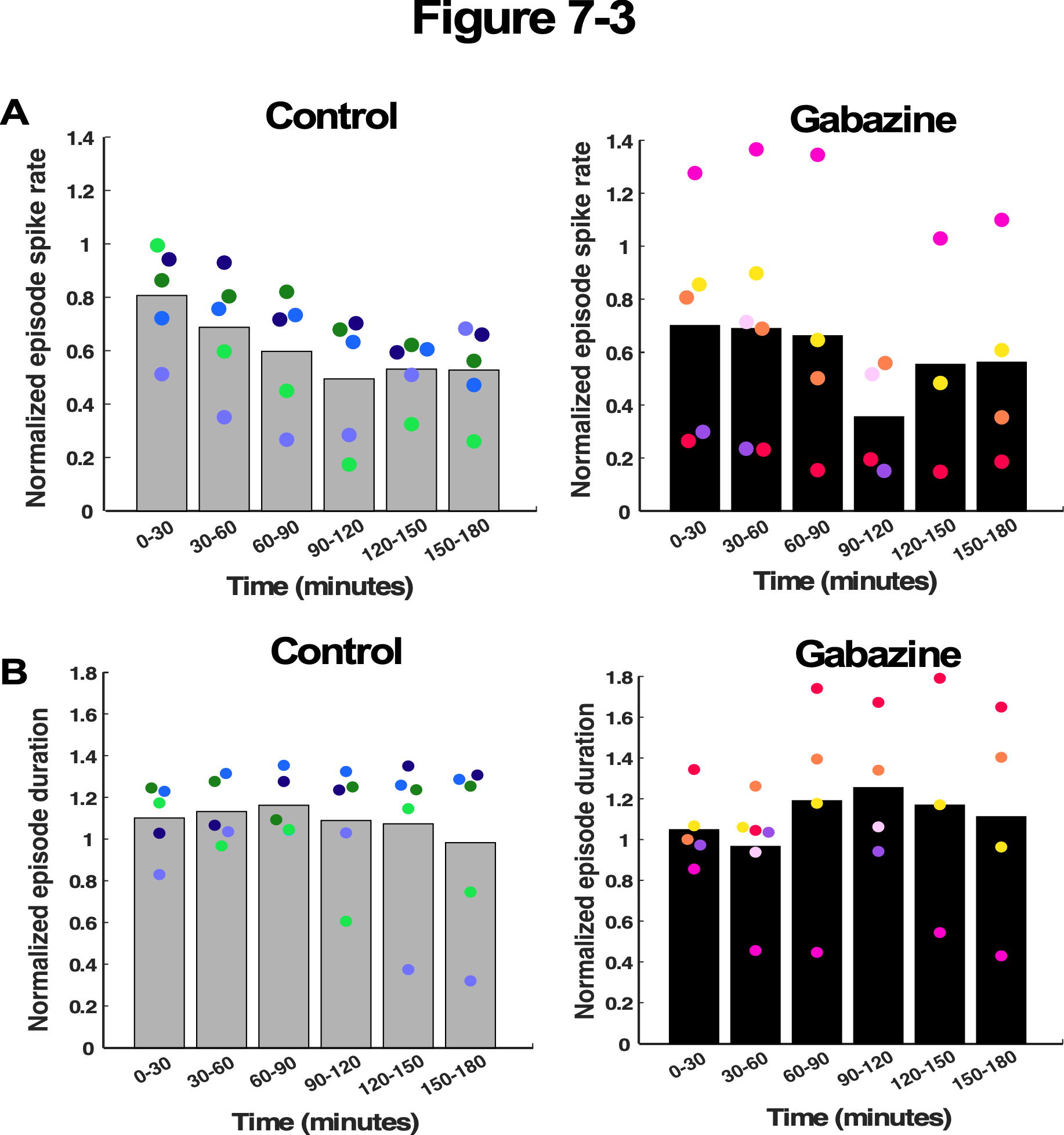

Supplement: Figure 7-3 — Episode spike rate and episode duration following GABAR blockade were variable in both control and gabazine-treated spinal cords. A) Episode spike rate displayed over a 3-hour period for control (untreated) and gabazine-treated cords. Values in each 30-minute bin are normalized to baseline condition. Each color dot represents a single spinal cord, with the height of the bar representing the mean of all cords. B) Episode duration displayed over a 3-hour period for control (untreated) and gabazine-treated cords. Values in each 30-minute bin are normalized to baseline condition. Each color dot represents a single spinal cord, with the height of the bar representing the mean of all cords. Download Fig 7-3, TIF file. [file eneuro-11-ENEURO.0259-24.2024-s008.tif]
